# Identification of FDA-approved antivirulence drugs targeting the *Pseudomonas aeruginosa* quorum sensing effector protein PqsE

**DOI:** 10.1080/21505594.2020.1770508

**Published:** 2020-05-28

**Authors:** Valerio Baldelli, Francesca D’Angelo, Viola Pavoncello, Ersilia Vita Fiscarelli, Paolo Visca, Giordano Rampioni, Livia Leoni

**Affiliations:** a Department of Science, University Roma Tre, Rome, Italy; b Laboratory of Cystic Fibrosis Microbiology, Bambino Gesú Hospital, Rome, Italy

**Keywords:** *Pseudomonas aeruginosa*, antivirulence strategy, quorum sensing inhibition, nitrofurazone, erythromycin estolate, screening, PqsE

## Abstract

The ability of the bacterial pathogen *Pseudomonas aeruginosa* to cause both chronic and acute infections mainly relies on its capacity to finely modulate the expression of virulence factors through a complex network of regulatory circuits, including the *pqs* quorum sensing (QS) system. While in most QS systems the signal molecule/receptor complexes act as global regulators that modulate the expression of QS-controlled genes, the main effector protein of the *pqs* system is PqsE. This protein is involved in the synthesis of the QS signal molecules 2-alkyl-4(1*H*)-quinolones (AQs), but it also modulates the expression of genes involved in virulence factors production and biofilm formation *via* AQ-independent pathway(s). *P. aeruginosa pqsE* mutants disclose attenuated virulence in plant and animal infection models, hence PqsE is considered a good target for the development of antivirulence drugs against *P. aeruginosa*.

In this study, the negative regulation exerted by PqsE on its own transcription has been exploited to develop a screening system for the identification of PqsE inhibitors in a library of FDA-approved drugs. This led to the identification of nitrofurazone and erythromycin estolate, two antibiotic compounds that reduce the expression of PqsE-dependent virulence traits and biofilm formation in the model strain *P. aeruginosa* PAO1 at concentrations far below those affecting the bacterial growth rate. Notably, both drugs reduce the production of the PqsE-controlled virulence factor pyocyanin also in *P. aeruginosa* strains isolated from cystic fibrosis patients, and do not antagonize the activity of antibiotics commonly used to treat *P. aeruginosa* infection.

## Introduction

The spread of antibiotic resistance in bacterial pathogens is increasing at an unprecedented pace. While the mortality due to antibiotic-resistant infections rises worldwide, the antibiotic discovery pipeline is running dry, with few approvals of new antibiotics for human therapy in the last decades [1]. Consequently, new antibacterial agents active against antibiotic-resistant pathogens are urgently needed. However, traditional antibiotic research programs seem unable to cope with the rapid spread of antibiotic resistance, mainly due to the high costs and long times required for *de novo* drug-discovery [2–4].

In the last years, the repurposing of “old” drugs for new clinical applications has become a major research area in drug discovery. In principle, the identification of off-target activity in drugs already approved for their use in humans allows fast and cost-effective selection of safe drugs with high potential for seamless adoption into the clinical practice [5,6]. The search for drugs targeting the growth and/or viability of bacterial pathogens remains a primary goal, but additional approaches to combat bacterial infections should be pursued in parallel. In this context, a promising antibacterial strategy aims at identifying molecules targeting bacterial virulence rather than bacterial growth or viability. The antivirulence approach has been boosted by increased knowledge on bacterial pathobiology, and it is expected to reduce bacterial adaptability to the host environment while posing a reduced selective pressure for the emergence of resistance relative to antibiotics. Moreover, by inhibiting pathogen-specific targets, antivirulence drugs could be endowed with limited adverse effects on the host microbiota [7–9].

The versatile Gram-negative bacterium *Pseudomonas aeruginosa* is able to colonize a variety of harsh environments, including polluted soil and marine habitats, plants and mammalian tissues [10]. As a human pathogen, *P. aeruginosa* has evolved a number of mechanisms for adaptation and survival within the host, including intrinsic and acquired resistance to multiple classes of antibiotics [10,11]. In particular, antibiotic-resistant biofilms are a major cause of hard to treat *P. aeruginosa* infections, mainly in healthcare settings, and the leading cause of morbidity and mortality in cystic fibrosis (CF) patients. CF is a genetic disease affecting ca. 1/3,000 newborns in the Caucasian population [12,13]. For these reasons, *P. aeruginosa* is included in the priority list of pathogens for which new antimicrobial therapies are urgently needed (Priority 1: Critical; http://www.who.int/en/news-room/detail/27-02-2017-who-publishes-list-of-bacteria-for-which-new-antibiotics-are-urgently-needed).

*P. aeruginosa* produces an array of toxic metabolites and enzymes, and different macromolecules contributing to the biofilm matrix [10]. Numerous efflux pumps and secretion systems contribute to the dangerous armament of this tough microorganism [14,15]. Finally, multiple interwoven global regulatory systems coordinate the expression of *P. aeruginosa* virulent phenotypes in response to population density and environmental cues [16,17]. Indeed, *P. aeruginosa* ability to colonize different human tissues, and to resist to the immune system and to antibiotics mainly relies on its capacity to finely modulate the expression of multiple virulence factors and to form biofilms [18,19]. For these reasons, *P. aeruginosa* global regulatory systems, including the quorum sensing (QS) circuits, are considered valuable targets for the development of antivirulence drugs [9,20,21].

*P. aeruginosa* has three major QS systems, namely the *las, rhl* and *pqs* systems. The *las* and *rhl* QS systems are based on acyl-homoserine lactones (AHLs), while the *pqs* QS system is based on 2-alkyl-4(1*H*)-quinolones (AQs) as signal molecules [19,22]. *P. aeruginosa* QS-deficient mutants display attenuated virulence in different animal models of infection, and for this reason QS is considered a good target for the development of *P. aeruginosa* antivirulence drugs [16,20,21,23]. However, the use of QS inhibitors for CF therapy is debated, mainly as a consequence of frequent isolation of *P. aeruginosa* mutants inactivated in the *las* QS system from CF patients with late chronic infection [24–27]. Conversely, the highest proportion of *P. aeruginosa* strains isolated from CF patients are AQ-producers [28,29], and AQ levels correlate with the clinical status of CF patients infected by *P. aeruginosa* [30], indicating that the *pqs* QS system could be a suitable target for innovative CF therapies.

The main AQ signal molecules of *P. aeruginosa* are 2-heptyl-3-hydroxy-4(1*H*)-quinolone (PQS) and its precursor 2-heptyl-4-hydroxyquinoline (HHQ). AQs production increases during bacterial growth, and when the level of these molecules in the environment reaches a threshold concentration, corresponding to the “*quorum*” cell density, either HHQ or PQS signal molecules bind to and activate the transcriptional regulator PqsR (also known as MvfR). The PqsR/AQ complex activates the transcription of the *pqsABCDE-phnAB* operon, coding for the enzymes required for the synthesis of HHQ, hence triggering the positive feedback loop typical of all QS systems. The *pqsH* gene codes for the PqsH enzyme required to convert HHQ to PQS [31–33]. While in the majority of bacterial QS systems the signal molecule/receptor complex acts as a global regulator to modulate the expression of QS genes, the main effector protein of the *pqs* system is PqsE rather than the PqsR/AQ complex. Indeed, data produced in our laboratory indicate that the main physiological role of the PqsR/AQ complex is to trigger transcription of the *pqsABCDE-phnAB* operon, ultimately resulting in increased production of HHQ and expression of PqsE, a thioesterase coded by the fifth gene of the *pqsABCDE-phnAB* operon [34]. PqsE is involved in AQ synthesis by converting 2-aminobenzoylacetyl-CoA into 2-aminobenzoylacetate [33], that is in turn condensed with octanoyl-coenzyme A by the PqsBC heterodimer to form HHQ [31]. However, *pqsE* inactivation does not significantly affect AQs biosynthesis [35,36], likely because PqsE thioesterase activity can be provided by alternative enzymes [33]. Intriguingly, PqsE controls the transcription of more than 140 genes, including key virulence genes, *e.g*. genes required for pyocyanin and rhamnolipids production, and genes involved in swarming motility and biofilm formation, *via* a still poorly understood pathway that is both PqsR- and AQs-independent [34–41]. Accordingly, *P. aeruginosa pqsE* mutants are impaired in biofilm formation and display reduced virulence in plant and animal models of infection [35,36,40]. Since PqsE expression requires the PqsR/AQs complex, PqsR-inhibitors have been shown to attenuate *P. aeruginosa* virulence both *in vitro* and in animal models of infection [42–52], and some of these inhibitors potentiate the effect of antibiotics used in CF therapy both *in vitro* and in murine models of infection [46,48]. To the best of our knowledge, only one PqsE inhibitor has been described so far. This molecule hampers PqsE thioesterase activity, but it does not affect PqsE ability to control the expression of virulence factors [39].

Interestingly, PqsE regulates its own expression. Indeed, the activity of the *pqsABCDE-phnAB* promoter P*pqsA* increases in the absence of PqsE, and is abrogated in a *P. aeruginosa pqsE*-overexpressing strain, suggesting that the PqsE protein directly or indirectly represses the *pqsABCDE-phnAB* operon [35,36]. In this study, the PqsE-dependent negative feedback loop has been exploited to develop a high-throughput screening system for the identification of molecules targeting PqsE activity. The screening system was validated by screening a library of 1,600 FDA-approved drugs, leading to the identification of anti-PqsE activity in nitrofurazone and erythromycin estolate. Although endowed with antibacterial properties, both compounds displayed antivirulence activity at concentrations far below those inhibiting *P. aeruginosa* growth. The effect of both drugs on the expression of *P. aeruginosa* virulence phenotypes, on biofilm formation and on the susceptibility to antibiotics currently used to treat *P. aeruginosa* infection has been investigated, as well as their activity against *P. aeruginosa* CF isolates. Beside their possible development as antivirulence agents, the new PqsE inhibitors identified in this study could facilitate future understanding of the molecular mechanism underlying PqsE-dependent control of virulence traits in *P. aeruginosa*.

## Materials and methods

### Bacterial strains, media and chemicals

The bacterial strains and plasmids used in this study are listed in Table S1 and Table S2, respectively. *Escherichia coli* DH5αF’ [53] was used for plasmid DNA amplification. Plasmids purification from *E. coli* and transformation into *P. aeruginosa* were performed with standard procedures [54].

All *E. coli* and *P. aeruginosa* strains were routinely grown at 37°C in Lysogeny Broth (LB) [54] with aeration. For some experiments, *P. aeruginosa* was grown in M9 minimal medium supplemented with 20 mM glucose as sole carbon source (M9-Glu) [54] or in BBL Mueller Hinton II Broth (Cation-Adjusted) medium (MHB, Becton Dickinson). The following antibiotics were added when required: 100 µg/mL ampicillin (Ap), for *E. coli*; 400 µg/mL carbenicillin (Cb) or 200 µg/mL tetracycline (Tc), for *P. aeruginosa*. When necessary, isopropyl β-D-1-thiogalactopyranoside (IPTG) was added at the concentrations indicated in the text. Stock solutions of 80 mM nitrofurazone (Fluka), 200 mM erythromycin estolate (Sigma-Aldrich) and 80 mM diminazene (Sigma-Aldrich) were prepared in dimethyl sulfoxide (DMSO), ethanol (EtOH) and water, respectively.

### Primary screening for the identification of PqsE inhibitors

The *P. aeruginosa* PqsE-Rep biosensor strain (*i.e., P. aeruginosa* PAO1 *pqsE*^IND^ P*pqsA::luxCDABE* [36]) was grown overnight at 37°C on LB agar plates. Bacteria were scraped from plates surface and diluted in LB supplemented with 50 µM IPTG to an optical density at 600 nm wavelength (OD_600_) of 0.08 (procedure modified from [55]). Two-hundred µL aliquots of the culture were grown at 37°C in 96-well black clear-bottom microtiter plates in the presence of compounds of the PHARMAKON library (20 µM and 200 µM). The OD_600_ and relative light units (RLU) were measured after 5 h incubation by using a Spark 10 M multilabel plate reader (Tecan). Samples grown in the presence of the solvent vehicle DMSO [0.2% (v/v) or 2% (v/v)] were used as controls in each microtiter plate. Reporter activity was determined as RLU/OD_600_ for each sample. Residual reported activity was determined in treated samples relative to the solvent vehicle control samples grown in the presence of DMSO, considered as 100%.

### Measurements of promoter activity

Bioluminescence was determined as a function of population density by using a Spark 10 M multilabel plate reader (Tecan), as previously described [34]. Briefly, overnight cultures of the *P. aeruginosa* reporter strains used in this study (Table S1) were diluted in 200 µL of LB to an OD_600_ ≈ 0.01, in the presence or in the absence of PqsE inhibitors at the concentrations indicated in the text, and dispensed into 96-wells black clear-bottom microtiter plates. When required, LB was supplemented with IPTG, at the concentrations indicated in the text. Luminescence and turbidity were determined after 5 h of incubation at 37°C with gentle shacking (120 rpm). Reporter activity was determined as RLU/OD_600_ for each sample. Residual reported activity was determined in treated samples relative to the solvent vehicle control samples grown in the presence of DMSO or EtOH, considered as 100%. The EC_50_ values (µM) were determined as the drugs concentration able to enhance P*pqsA::lux* activity of 50% with respect to the untreated control.

### Analyses of virulence-related phenotypes

For the *P. aeruginosa* PqsE-Rep, PAO1 wild type and PAO1 ∆*pqsE* strains, pyocyanin was extracted and quantified as previously described [56]. The same method was scaled-down in order to extract and quantify pyocyanin produced by *P. aeruginosa* CF isolates. Briefly, each CF strain was incubated in 96-wells microtiter plates for 21 h at 37°C with shacking (120 rpm) in 200 µL of LB broth, in the presence of 100 µM nitrofurazone or 50 µM erythromycin estolate. Each CF strain was incubated in the presence of 0.125% (v/v) DMSO or 0.025% (v/v) EtOH as solvent vehicle control (untreated samples). After 21 h incubation, two independent cultures of the same CF strain were pooled, the OD_600_ was measured and cell-free supernatants were collected into 1.5 mL tubes. After extraction with an isovolume of chloroform, the pyocyanin-containing chloroform phase was transferred into clean 1.5 mL tubes and acidified with an isovolume of 0.2 N HCl. After centrifugation, 200 µL of the aqueous-phase were transferred into 96-wells microtiter plates and A_520_ was measured by using an automated Spark 10 M multilabel plate reader (Tecan). Pyocyanin production was evaluated by normalizing the A_520_ to the OD_600_ value measured for each CF strain. The IC_50_ values (µM) were determined as the drugs concentration able to decrease pyocyanin production of 50% with respect to the untreated control.

Rhamnolipids in cell-free supernatants of *P. aeruginosa* cultures were quantified by the orcinol method, as previously described [57]. Briefly, bacterial strains were grown at 37°C for 24 h in LB supplemented with 100 µM nitrofurazone, 50 µM erythromycin estolate, 0.125% (v/v) DMSO or 0.025% (v/v) EtOH before rhamnolipids extraction and quantification.

For swarming motility assay, 5 µL of *P. aeruginosa* cultures grown in LB for 8 h were spotted onto swarming plates [0.8% (w/v) nutrient broth N.2, 0.5% (w/v) glucose, 0.5% (w/v) bacteriological agar] supplemented with 100 µM nitrofurazone or 50 µM erythromycin estolate. Also in this case, plates supplemented with the solvent vehicles 0.125% (v/v) DMSO or 0.025% (v/v) EtOH were used as controls. After 16 h of growth at 37°C, swarming motility was directly observed at the air-agar interface [58].

For microscopic visualization of biofilm, *P. aeruginosa* strains constitutively expressing GFP *via* the pMRP9-1 plasmid [59] were grown in 8-well chamber slides, as previously described [60], with minor modifications. Briefly, bacteria were inoculated at an OD_600_ of 0.02 in 500 µL of M9-Glu in the presence and in the absence of the tested compounds (*i.e*., 100 µM nitrofurazone, 50 µM erythromycin estolate, 0.125% (v/v) DMSO or 0.025% (v/v) EtOH), and incubated at 30°C for 72 h. To maintain bacterial viability, the medium was changed every 24 h. The biofilm structure was examined using a Leica TCS SP5 confocal microscope.

### MIC and antibiotic tolerance assays

The Minimal Inhibitory Concentration (MIC) of the antibiotics ciprofloxacin, colistin, tobramycin and piperacillin was evaluated with the standard microdilution method, according to the Clinical and Laboratory Standards Institute guidelines [61]. Briefly, *P. aeruginosa* PAO1 and its isogenic ∆*pqsE* mutant were grown at 37°C with shaking in MHB or in M9-Glu [53]. After 16 h of growth, the cultures were diluted in 100 µL of MHB or M9-Glu to an OD_600_ ≈ 0.0005 (ca. 5 × 10^5^ CFU/mL) in 96-well microtiter plates with increasing concentrations of the selected antibiotics. For tobramycin, possible interaction with 100 µM nitrofurazone or 50 µM erythromycin estolate was also evaluated. The MIC values were evaluated after 24 h of static incubation at 37°C.

The fraction of tolerant *P. aeruginosa* cells was determined as previously described [46]. Briefly, *P. aeruginosa* strains were grown with shaking and aeration to mid-logarithmic phase in LB, in the absence or in the presence of 100 µM nitrofurazone or 50 µM erythromycin estolate [0.125% (v/v) DMSO or 0.025% (v/v) EtOH were used as solvent vehicle controls]. Before antibiotic addition, culture aliquots were diluted in fresh LB and plated on LB agar for CFU count (pre-antibiotic). The rest of the culture was treated with 4 µg/mL tobramycin (8x MIC). At 16 h post-antibiotic addition, culture aliquots were washed twice in fresh LB to remove antibiotic carry-over, serially diluted and plated on LB agar for CFU count. This procedure was repeated at 24 h post-antibiotic addition to ensure that a killing plateau was reached. The tolerant fraction expressed as N-fold change was determined as the ratio between the CFU/mL values measured after antibiotic addition (16 h and 24 h post-antibiotic) divided by CFU/mL values measured before antibiotic addition.

### Statistical analysis

Statistical analysis was performed with the software GraphPad Prism 5, using one-way analysis of variance (ANOVA) followed by Tukey-Kramer multiple comparison tests. Differences having a *p* value < 0.05 were considered statistically significant.

## Results

### Development and validation of a screening system for the identification of PqsE inhibitors

**Figure 1.**
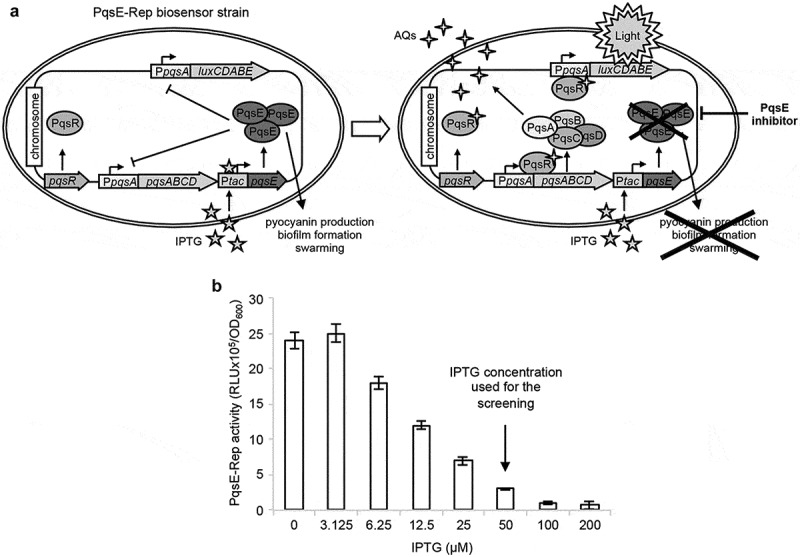
Screening system developed for the identification of PqsE inhibitors. (a) Schematic representation of the PqsE-Rep-based reporter system. The PqsE-Rep strain contains the P*pqsA::lux* transcriptional fusion and a genetic cassette for IPTG-inducible expression of the *pqsE* gene. Since in *P. aeruginosa* PqsE represses P*pqsA* promoter activity, the PqsE-Rep biosensor emits light at basal level when grown in LB supplemented with IPTG; as a consequence, molecules affecting PqsE functionality are expected to increase light emission by P*pqsA* derepression. (b) Activity of the P*pqsA* promoter in the PqsE-Rep strain grown in LB supplemented with the indicated concentrations of IPTG. PqsE-Rep was inoculated at an OD_600_ of 0.08 in 0.2 mL of LB in 96-well microtiter plates and light emission was measured after 5 h of incubation at 37°C in shaking conditions. The average of three independent experiments is reported with SD.

In this study the *P. aeruginosa* PAO1 derivative strain previously named *P. aeruginosa pqsE*^IND^ P*pqsA::lux* [36] has been re-named PqsE-Rep. This recombinant strain expresses the *pqsE* gene under the control of the IPTG-inducible P*tac* promoter and carries a transcriptional fusion between the promoter of the *pqsABCDE-phnAB* operon (P*pqsA*) and the *luxCDABE* operon for bioluminescence emission integrated at the neutral *attB* site of the chromosome (Figure 1(a)). A previous study showed that the IPTG-dependent induction of *pqsE* expression decreases bioluminescence emitted by PqsE-Rep, and that IPTG *per se* does not affect P*pqsA* activity in wild type *P. aeruginosa* PAO1 up to a concentration of 1 mM [36]. These data indicate that the PqsE-Rep biosensor strain could be exploited as a reporter system to identify inhibitors of PqsE activity. Preliminary experiments were carried out to select culture conditions optimal for carrying out a screening campaign aimed at identifying PqsE inhibitors by using the PqsE-Rep biosensor strain (Figure S1). The bioluminescence emitted by PqsE-Rep inversely correlated with the amount of IPTG present in the medium (Figure 1(b)). This is because the increase in IPTG concentration induces PqsE expression, leading to a parallel PqsE-dependent repression of P*pqsA* activity. In the presence of IPTG, the addition of a PqsE inhibitor to the PqsE-Rep biosensor strain should result in a significant increase of bioluminescence with respect to the untreated control. Since 50 µM IPTG strongly decreased P*pqsA* activity without saturating PqsE-Rep response (Figure 1(b)), this IPTG concentration was used throughout the screening campaign.

To identify new PqsE inhibitors, the PqsE-Rep reporter system was used to screen the PHARMAKON library of 1,600 FDA-approved compounds with known biological activity and high chemical and pharmacological diversity. Both 20 µM and 200 µM concentrations of each compound were used to detect any increase in bioluminescence emission by PqsE-Rep relative to the untreated control (details in Materials and Methods). Considering bioluminescence and cell density of the solvent vehicle control samples as 100%, the criteria for hits selection were: *i*) bioluminescence ≥ 130% at 20 µM; *ii*) bioluminescence ≥ 200% at 200 µM; *iii*) reduction of cell density ≤ 10% at both 20 µM and 200 µM. This primary screen led to the selection of 24 hits (Figure S2A).

**Figure 2.**
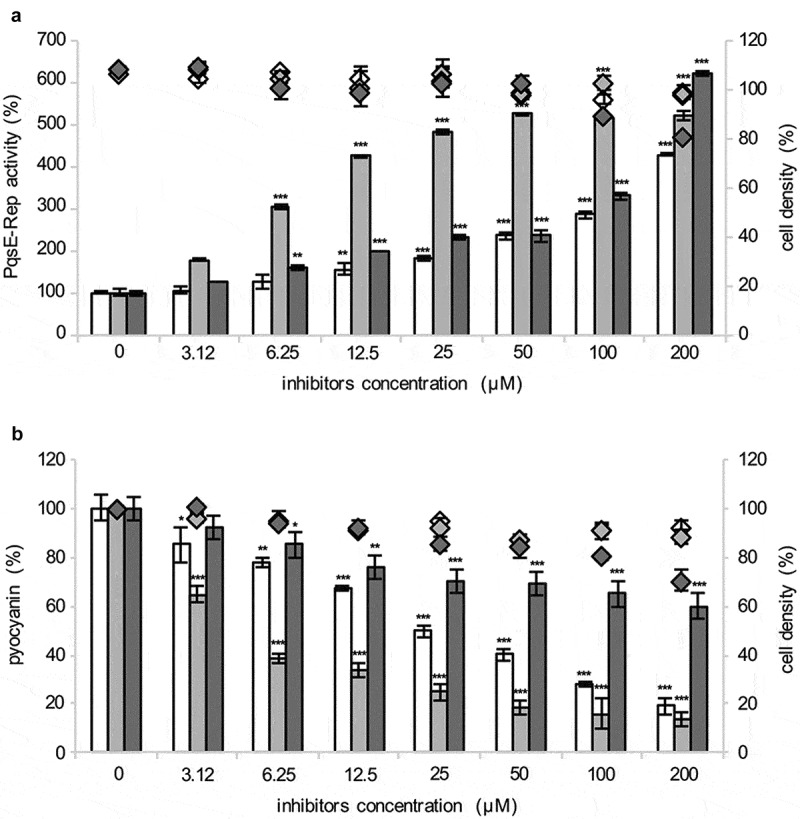
Selected hits increase P*pqsA* activity and reduce pyocyanin production. Effect of nitrofurazone (white bars), erythromycin estolate (light-gray bars) and diminazene aceturate (dark-gray bars) on PqsE-Rep bioluminescent emission (a) and pyocyanin production (b). Solvent vehicle control samples were considered as 100%. The average of at least three independent experiments is reported with SD. Statistical significance relative to the untreated control is indicated with asterisks: *, *p* < 0.05; **, *p* < 0.005; ***, *p* < 0.001 (ANOVA).

Since the production of the PqsE-dependent virulence factor pyocyanin parallels IPTG-dependent induction of PqsE in the PqsE-Rep biosensor strain [36], the effect of the 24 hits on pyocyanin production was used as a proxy in a secondary screening aimed at deselecting hits increasing PqsE-Rep bioluminescence emission *via* PqsE-independent mechanisms. Out of the 24 selected hits, only nitrofurazone, erythromycin estolate and diminazene aceturate robustly reduced pyocyanin production in PqsE-Rep grown in the presence of 50 µM IPTG compared with the solvent vehicle control samples, without affecting bacterial growth (Figure S2B). These three compounds were purchased from different providers, and their efficacy in increasing light emission and decreasing pyocyanin production was tested again in the PqsE-Rep strain grown in the presence of 50 µM IPTG. Diminazene aceturate was still able to increase light emission (Figure 2(a)), but it showed low activity as a pyocyanin inhibitor (Figure 2(b)). Conversely, nitrofurazone and erythromycin estolate increased light emission (Figure 2(a)) and strongly decreased pyocyanin production in a dose dependent manner (Figure 2(b)). EC_50_ and IC_50_ values were 78.58 µM and 24.65 µM for nitrofurazone, and of 6.15 µM and 5.79 µM for erythromycin estolate, respectively (Table 1). Hence, nitrofurazone and erythromycin estolate were selected for further investigations.

**Table 1.** PqsE inhibitors identified by screening the PHARMAKON library of FDA-approved drugs.

Drug name	Property	Structure	EC_50_^a^	IC_50_^a^
nitrofurazone	antibacterial		78.58	24.65
erythromycin estolate	antibacterial		6.15	5.79

^a^The EC_50_ and IC_50_ values (µM) are relative to the ability of the drugs to enhance P*pqsA::lux* activity and to inhibit pyocyanin production, respectively, determined by using the PqsE-Rep reporter strain.

### Nitrofurazone and erythromycin estolate inhibit the expression of PqsE-controlled virulence phenotypes

Nitrofurazone and erythromycin estolate are antibiotics belonging to the nitrofuran and macrolide structural classes, respectively [62,63]. However, the MIC of these drugs for *P. aeruginosa* PAO1 and for its isogenic ∆*pqsE* mutant is ≥ 3.2 mM (see Materials and Methods). Neither nitrofurazone, nor erythromycin estolate decreased the bacterial growth rate in LB at the concentrations used in this study (Fig. S3), as expected for antivirulence drugs.

**Figure 3.**
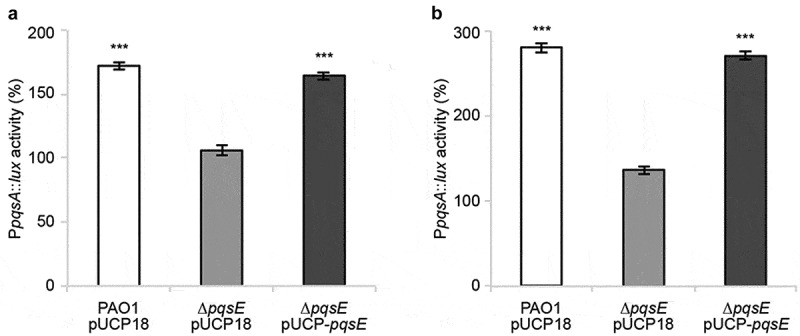
Nitrofurazone and erythromycin estolate increase P*pqsA* activity only in a *pqsE*-proficient background. Effect of 100 µM nitrofurazone (a) or 50 µM erythromycin estolate (b) on P*pqsA* promoter activity in the indicated strains. Promoter activity is reported as percentage with respect to the corresponding solvent vehicle control sample, considered as 100%. The average of three independent experiments is reported with SD. Statistical significance relative to the untreated control is indicated with asterisks: ***, *p* < 0.001 (ANOVA).

The PqsE-Rep biosensor used to select nitrofurazone and erythromycin estolate is an engineered strain in which *pqsE* expression is driven by the heterologous P*tac* rather than by the native P*pqsA* promoter. To validate the PqsE-dependent regulatory network as a target for nitrofurazone and erythromycin estolate, the effect of these drugs on P*pqsA* activity was evaluated by means of a P*pqsA::luxCDABE* transcriptional fusion in wild type *P. aeruginosa* PAO1 and in its isogenic ∆*pqsE* mutant. As shown in Figure 3, nitrofurazone and erythromycin estolate increased P*pqsA* activity in *P. aeruginosa* PAO1 by 172.1% and 280.1% relative to the control samples (solvent vehicle), respectively. This effect was abolished in the ∆*pqsE* mutant and restored by *in trans* expression of the *pqsE* gene. Control experiments performed in the same genetic backgrounds carrying chromosomal integration of the empty vector mini-CTX-*lux* [64], in which light emission does not rely on P*pqsA* activity or PqsE functionality, revealed that nitrofurazone and erythromycin estolate do not affect the bioluminescent emission driven by the vector (Fig. S4).

**Figure 4.**
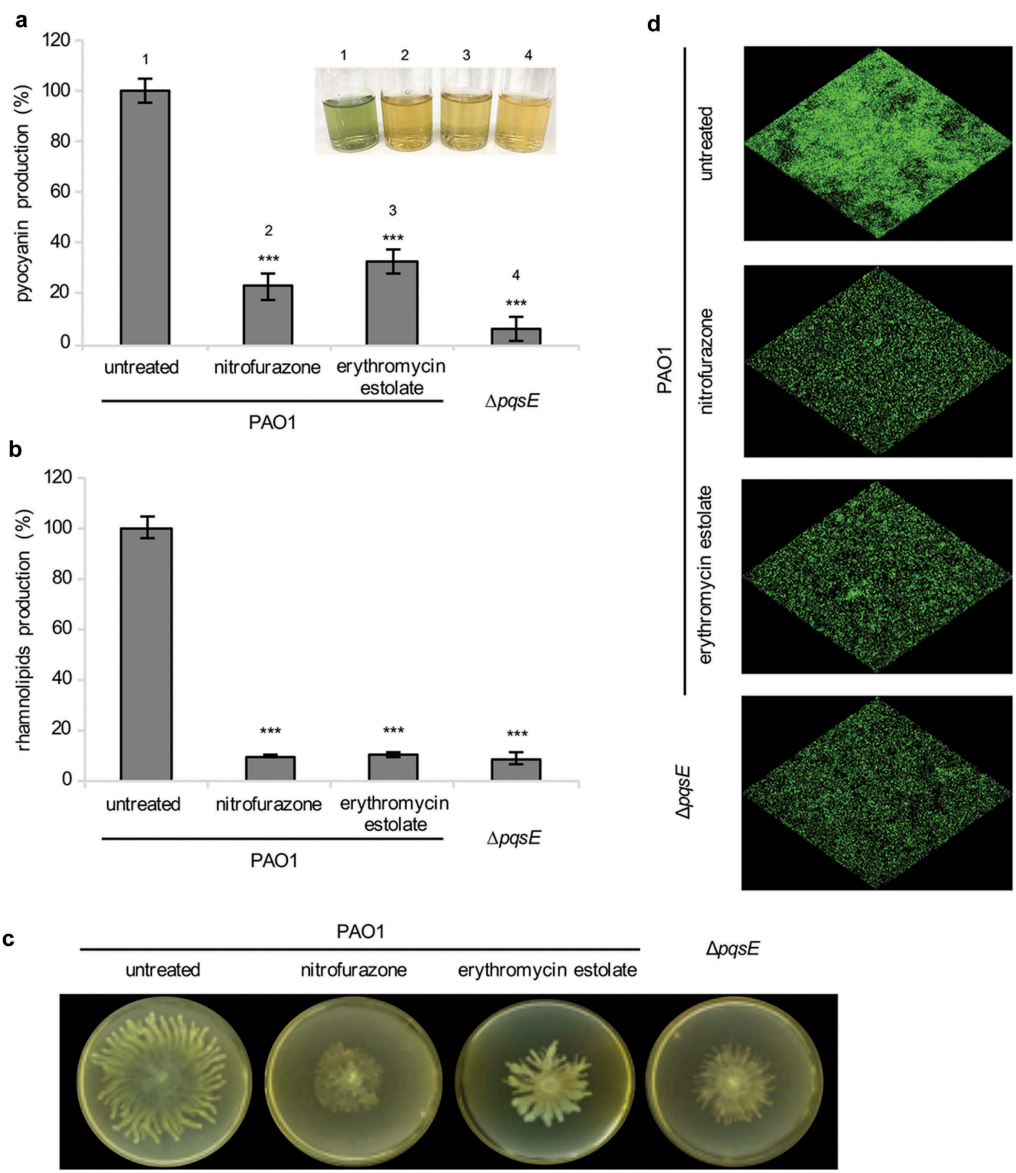
Nitrofurazone and erythromycin estolate inhibit the expression of PqsE-controlled virulence traits. Effect of 100 µM nitrofurazone or 50 µM erythromycin estolate on pyocyanin (a) and rhamnolipids (b) production, on swarming motility (c) and on biofilm formation (d) in *P. aeruginosa* PAO1. The isogenic ∆*pqsE* mutant was used as a control. For pyocyanin and rhamnolipids production, the average of three independent experiments is reported with SD; representative supernatants are shown in the inset picture in **(A)**. Statistical significance relative to the untreated control is indicated with asterisks: ***, *p* < 0.001 (ANOVA). For swarming motility and biofilm formation, one representative picture of three independent experiments is shown.

Notably, nitrofurazone and erythromycin estolate strongly reduced PqsE-dependent virulence traits in wild type *P. aeruginosa* PAO1, including pyocyanin and rhamnolipids production, and swarming motility, thus mimicking *pqsE* deletion (Figure 4(a-c) [35–37,65]. Conversely, none of these drugs affected the PqsE-independent surface motilities twitching and swimming (data not shown) [36].

The effect of PqsE inactivation on *P. aeruginosa* biofilm formation was tested by confocal microscopy analysis, using wild type PAO1 and its isogenic ∆*pqsE* mutant constitutively expressing GFP. In line with literature data [36], *pqsE* mutation reduced the ability of PAO1 to form biofilm (Figure 4(d)). As expected for PqsE inhibitors, nitrofurazone and erythromycin estolate were able to decrease biofilm formation in wild type *P. aeruginosa* PAO1 (Figure 4(d)) without affecting cell density of the planktonic phase (data not shown). These results suggest that the ability of nitrofurazone and erythromycin estolate to decrease biofilm formation could depend on their anti-PqsE activity, and likely on the consequent reduction in pyocyanin and rhamnolipids levels. Overall, these results support the PqsE-dependent regulatory circuitry as a target for nitrofurazone and erythromycin estolate.

### Effect of nitrofurazone and erythromycin estolate in combination with antibiotics

The transfer of a new anti-*Pseudomonas* drug to the clinical practice requires the assessment of its possible interaction with existing therapies. Moreover, antivirulence drugs targeting PqsR have been shown to potentiate the effect of some antibiotics clinically used to treat *P. aeruginosa* infection, *e.g*. tobramycin and ciprofloxacin [46,48]. In a preliminary analysis, the effect of *pqsE* deletion on *P. aeruginosa* MICs for tobramycin, ciprofloxacin, piperacillin and colistin was determined according to the standard microdilution procedure using both Mueller Hinton Broth (MHB), and M9 glucose minimal medium (M9-Glu) that had been employed for the biofilm assay. Results showed that *pqsE* deletion does not affect the MIC for ciprofloxacin, piperacillin and colistin, irrespective of the growth medium (Table S3). Interestingly, in M9-Glu the MIC of tobramycin for the ∆*pqsE* mutant was 0.25 µg/mL, *i.e*. 2-fold lower than the MIC observed for wild type PAO1 (0.5 µg/mL), while MICs were identical for both strains in MHB (Table S3). This suggested that PqsE-inhibitors could increase the susceptibility of *P. aeruginosa* PAO1 to tobramycin in M9-Glu medium. Intriguingly, the tobramycin MIC for wild type PAO1 was 4-fold reduced in the presence of nitrofurazone (*i.e*., from 0.5 µg/mL to 0.125 µg/mL), while it was unaffected by erythromycin estolate. Hence, nitrofurazone potentiates the activity of tobramycin against *P. aeruginosa* planktonic cultures grown in M9-Glu medium.

A previous study showed that drugs targeting PqsR can reduce the formation of *P. aeruginosa* antibiotic tolerant cells [46,48], hence we questioned whether PqsE inhibitors could disclose this same feature. Preliminary experiments carried out with tobramycin, ciprofloxacin, piperacillin and colistin showed the existence of a detectable fraction of antibiotic tolerant cells that remained constant 16 h and 24 h post-treatment only in *P. aeruginosa* PAO1 cultures treated with 4 µg/mL tobramycin (8 x MIC; data not shown). The fraction of tobramycin tolerant cells was unaffected by deletion of *pqsE* or by nitrofurazone treatment (Fig. S5). Surprisingly, erythromycin estolate increased of about 2-logs the amount of wild type PAO1 cells tolerant to tobramycin relative to the untreated control, likely *via* a PqsE-independent mechanism (Fig. S5).

### *Effect of nitrofurazone and erythromycin estolate on different* P. aeruginosa *isolates from CF patients*

Pyocyanin is a *P. aeruginosa* virulence factor important in CF lung infection [66], and its biosynthesis is under the control of the PqsE-dependent regulatory pathway [35–37,65]. Hence the ability of the PqsE-inhibitors nitrofurazone and erythromycin estolate to reduce pyocyanin production was preliminarily evaluated in a collection of 21 *P. aeruginosa* strains isolated from the lungs of CF patients. The CF isolates are all AQs and pyocyanin producers and can be evenly distributed in three categories with respect to the stage of infection (*i.e*., first isolation, early chronic or late chronic; Table 2).

**Table 2.** Effect of PqsE inhibitors on *P. aeruginosa* CF isolates.

Isolate name	Stage of infection^a^	Antibiotic susceptibility^b^	Pyocyanin residual production %	
			nitrofurazone ^c^	erythromycin estolate^d^
BG13	first isolation	MDR	80.5	11.1
BG30	first isolation	S	50.7	4.9
BG37	first isolation	R	48.8	1.4
BG45	first isolation	S	95.0	9.3
BG50	first isolation	S	91.6	2.1
BG69	first isolation	S	43.2	99.2
BG71	first isolation	S	73.4	99.0
BG14	early chronic	MDR	26.9	3.4
BG34	early chronic	R	19.2	5.9
BG38	early chronic	R	47.9	1.2
BG42	early chronic	R	79.2	9.3
BG46	early chronic	S	70.8	1.4
BG61	early chronic	S	22.9	13.2
BG6	late chronic	R	34.8	18.1
BG15	late chronic	MDR	61.2	39.1
BG29	late chronic	R	68.9	99.9
BG83	late chronic	R	35.1	68.5
BG89	late chronic	MDR	95.0	51.2
BG92	late chronic	MDR	99.7	75.0
BG93	late chronic	MDR	98.4	40.6
BG94	late chronic	MDR	54.7	63.4

**^a^** Different categories depending on the stage (years) of infection caused by the clinical isolate in the lung of cystic fibrosis patients: first isolate, early chronic (from 2 to 3 years); late chronic (more than 5 years).

**^b^** Criteria to define multi-drug resistant (MDR) bacteria according to the European Center for Diseases Prevention and Control (ECDC) web site (http://ecdc.europa.eu/en/Pages/home.aspx).

**^c^** Pyocyanin residual level in samples treated with 100 µM nitrofurazone expressed in % relative to solvent vehicle control samples [0.125% (v/v) DMSO], considered as 100%.

**^d^** Pyocyanin residual level in samples treated with 50 µM erythromycin estolate expressed in % relative to solvent vehicle control samples [0.025% (v/v) EtOH], considered as 100%.

**Figure 5.**
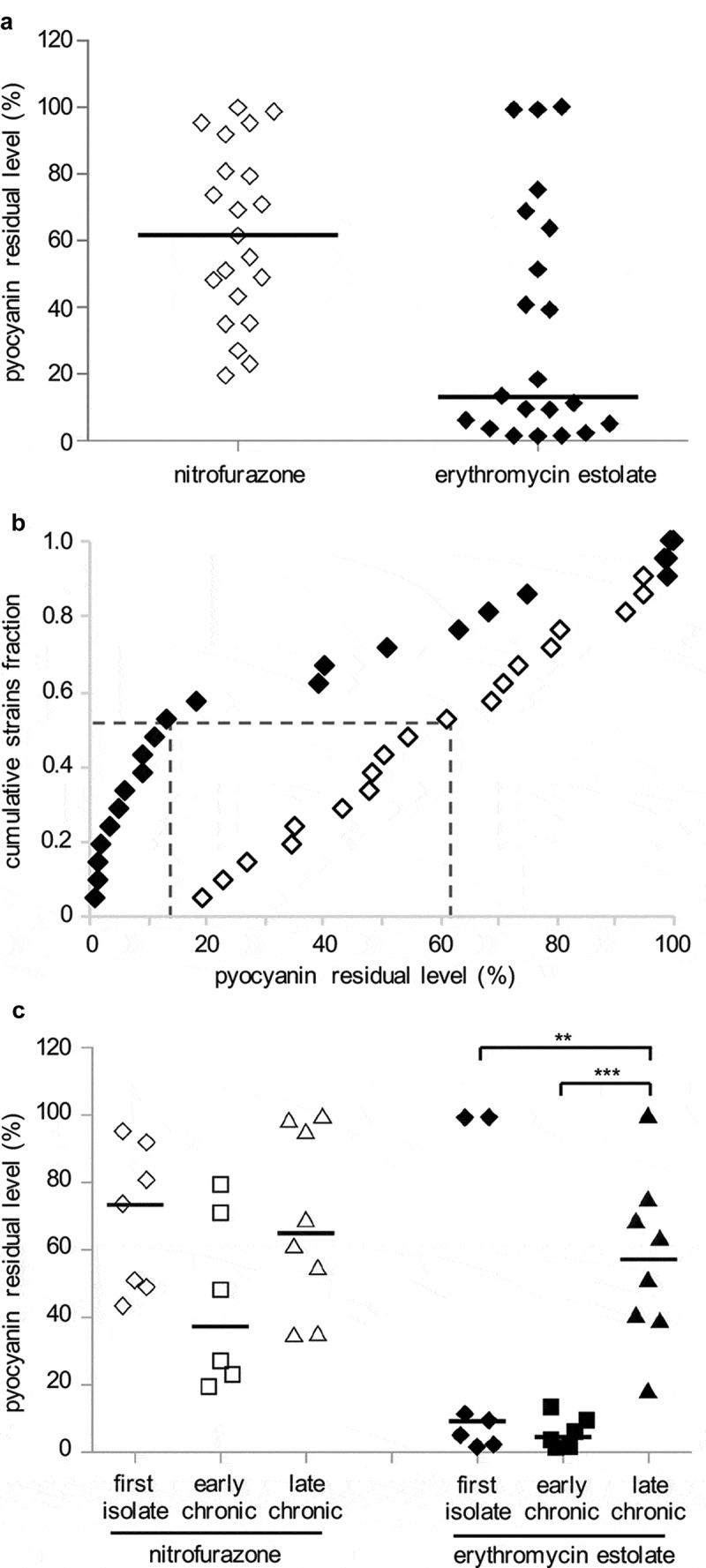
Nitrofurazone and erythromycin estolate are active against *P. aeruginosa* CF isolates. (a) Residual pyocyanin production in CF isolates grown in the presence of 100 μM nitrofurazone (white diamonds) or 50 µM erythromycin estolate (black diamonds) relative to solvent vehicle control samples, considered as 100%. Black lines represent the median values. The average of three independent experiments is reported. (b) Empirical cumulative distribution plots based on the data in (A). Differences between the distribution plots of nitrofurazone (white diamonds) and erythromycin estolate (black diamonds) are statistically significant (*p* < 0.001; ANOVA). Dashed lines indicate the residual pyocyanin production in 11 strains out of 21 (*cumulative strains fraction *= 0.524): ≤ 13.2% for erythromycin estolate and ≤ 61.2% for nitrofurazone. (c) Data from (A) clustered on the basis of the stage of infection: diamonds, CF strains isolated for the first time from patients; squares, CF strains isolated from patients with chronic infection from 2 to 3 years; triangles, CF strains isolated from patients with chronic infection for more than 5 years. **, *p* < 0.005; ***, *p* < 0.001 (ANOVA).

The 21 CF strains were grown in LB in the absence or in the presence of nitrofurazone or erythromycin estolate, and pyocyanin concentration was determined in the culture supernatants. Both nitrofurazone and erythromycin estolate decreased pyocyanin production of the CF strains to variable extents (Table 2 and Figure 5(a)), without affecting bacterial growth (data not shown). Overall erythromycin estolate showed a higher pyocyanin inhibitory activity compared to nitrofurazone. Indeed, the residual levels of pyocyanin produced by CF isolates treated with nitrofurazone were higher than those measured for erythromycin estolate-treated cultures, except for 5 out of 21 CF isolates (namely BG29, BG69, BG71, BG83 and BG94; Table 2). The higher inhibitory activity exerted by erythromycin estolate on pyocyanin production compared to nitrofurazone is also evident in the empirical distribution plot shown in Figure 5(b). In detail, this analysis highlighted that 11 out of 21 strains (*cumulative strain fraction *= 0.524) showed a residual pyocyanin production ≤ 13.2% and ≤ 61.2% when treated with erythromycin estolate and nitrofurazone, respectively (*p* < 0.01; Figure 5(b)).

An analysis carried out by clustering the CF isolates according to the stage of infection (expressed as years after primary isolation) showed that no significant correlation was found between stage of infection and susceptibility to nitrofurazone (Table 2 and Figure 5(c)). Conversely, CF first isolates and CF strains isolated from patients with early chronic infection (from 2 to 3 years) were significantly more susceptible to erythromycin estolate than the CF strains isolated from patients with late chronic infection (more than 5 years; Table 2 and Figure 5(c)). Indeed, 5 out of 7 strains isolated for the first time from a patient produced pyocyanin residual levels ≤ 11.1%, with the remaining 2 strains behaving as outliers (*i.e*., BG69 and BG71). The 6 strains isolated from patients with early chronic infection showed a similar behavior, producing pyocyanin residual levels ≤ 13.2%. Conversely, the residual level of pyocyanin was ≤ 51.2% in only 4 out of 8 CF isolates from patients with late chronic infection (Table 2 and Figure 5(c)).

## Discussion

*P. aeruginosa* is considered a model organism for QS and quorum quenching studies, due to the key role of its complex QS circuitry in pathogenicity. Indeed, the ability of this bacterium to adapt to the host environment and to cause infection relies on the fine-tuning of multiple virulence genes controlled by three major QS systems, *las, rhl* and *pqs*, whose expression and activity is strictly interwoven [22,67]. Recently, an additional QS system based on oxylipins as signal molecules and possibly required for full virulence has been identified in *P. aeruginosa*, increasing the complexity of its QS circuitry [68].

The *pqs* QS system controls the expression of multiple virulence factors and biofilm formation, as inferred by the evidence that *P. aeruginosa* mutants defective in the *pqs* QS system display attenuated pathogenicity in different plant and animal models of infection [35,36,40,41,46,69–75]. Although the central role played by the *pqs* system in *P. aeruginosa* pathobiology has been extensively studied, a clear characterization of the molecular mechanism by which its individual elements control gene expression is still unclear. DNA-protein interaction studies showed that the PqsR/AQ complex can bind to different promoter and intergenic regions [76,77]. However, a transcriptomic analysis carried out in a genetic background in which PQS production or PqsE expression were independent from PqsR/AQ activity revealed that the major effectors of the *pqs* system are PQS and PqsE, rather than the PqsR/AQ complex [34]. Indeed, in the absence of PqsR, PQS and PqsE control the expression of 179 and 145 genes, respectively, with an overlap of only 30 genes among the two regulons. Hence the main role played by the PqsR/AQ complex is to trigger the transcription of the *pqsABCDE-phnAB* operon, thus increasing the synthesis of PQS and the expression PqsE [34].

The main mechanisms underlying the effect of PQS on gene expression could be ascribed to its ability to interact with membranes and to chelate iron [34,78–81]. Despite the crystallographic structure of PqsE was determined more than ten years ago [82], the mechanism of action of this protein remains elusive. Briefly, PqsE can contribute to AQs biosynthesis *via* its thioesterase activity, but this function can be replaced by other thioesterases in a *pqsE*-depleted genetic background [33]. Moreover, molecules specifically targeting the PqsE thioesterase catalytic domain do not affect the expression of PqsE-dependent virulence factors, suggesting that PqsE is a multifunctional protein [39]. This hypothesis is supported by recent studies showing that PqsE is essential for the production of a molecule able to activate the LuxR-like receptor RhlR, encoded by the *rhlR* gene, in alternative to its cognate signal molecule *N*-butyryl-homoserine lactone (C_4_-HSL). Genetic data suggest that the complex between RhlR and this alternative ligand could trigger the expression of PqsE-dependent genes [40]. Notably, experiments carried out with a murine model of lung infection confirmed the central role played by PqsE in virulence. Indeed, a *P. aeruginosa* mutant unable to produce C_4_-HSL was as virulent as the wild type isogenic strain, while *pqsE* and *rhlR* mutant strains showed complete loss of virulence and attenuated virulence, respectively [40]. Finally, a recent work showed that a mutated variant of RhlR active in the absence of any ligand could only partially restore pyocyanin production in a *pqsE* mutant, also depending on the environmental conditions [41]. This study, together with the previous observation that the *pqsE* mutant was less virulent than the *rhlR* mutant *in vivo*, supports the hypothesis that PqsE could have multiple mechanisms of action besides being involved in AQs synthesis and being required for the production of the RhlR alternative ligand. It remains unclear whether PqsE is directly responsible for the production of the RhlR alternative ligand, or if intermediate factor(s) are involved in the PqsE-dependent activation of virulence genes. Overall, the current knowledge highlights the key role of PqsE as a hub in the complex regulatory QS circuitry required for full *P. aeruginosa* virulence, and supports PqsE as a target for antivirulence drugs.

Even if the mechanism of action of PqsE and the downstream regulative network affecting the expression of virulence genes remain largely unknown, the screening system described in this study was effective in identifying molecules specifically hampering the expression of PqsE-dependent virulence traits. Indeed, both nitrofurazone and erythromycin estolate were able to reduce the expression of PqsE-dependent phenotypes, including pyocyanin and rhamnolipids biosynthesis, swarming motility and biofilm formation. Moreover, nitrofurazone and erythromycin estolate relieved the PqsE-dependent repression of P*pqsA* in wild type *P. aeruginosa*. Notably, this latter activity was abrogated in a *pqsE*-deleted mutant and could be restored by genetic complementation.

Since the mode of action of PqsE is not fully understood, it is difficult to speculate about the mechanism(s) responsible for nitrofurazone- and erythromycin estolate-dependent PqsE inhibition. Nitrofuran drugs were extensively used in livestock production till 1995, when this use was banned in Europe and other countries due to concerns about the toxicity of their residues in edible animal tissues. However, some nitrofurans are currently used worldwide for the treatment of bacterial and protozoal infections in humans [83,84]. As an antibacterial agent, nitrofurazone is mainly used for topic application on wounds and catheters infected by both Gram-positive and Gram-negative pathogens. The nitrofurantoin mechanism of action is one of the best characterized among nitrofurans and the main process targeted by this antibiotic is mRNA translation, through oxidative damage to the ribosome [85,86]. Interestingly, a nitrofuran antibiotic named nifuroxazide was previously found to have anti-QS and antivirulence properties against *P. aeruginosa* [87]. It was noticed that the effect of nifuroxazide was stronger against *rhl-* and *pqs-* than against *las*-controlled virulence factors [87]. This observation, together with recent findings highlighting the strong connection between the *rhl* and *pqs* QS systems [40,41], supports the hypothesis that the main antivirulence activity of nitrofurans could be dependent on their ability to interfere with PqsE-controlled regulatory pathway(s).

Erythromycin is a macrolide antibiotic inhibiting bacterial growth by binding the 23S rRNA in the 50S subunit of the bacterial ribosome, thereby preventing the transfer of tRNA from the ribosome. Erythromycin estolate is almost fallen into disuse, replaced by less toxic and more effective macrolides [63]. It is important to highlight that macrolide antibiotics used at sub-MIC concentrations can inhibit *P. aeruginosa* virulence and biofilm formation [88]. The most striking example is azithromycin, which is used as adjuvant in the therapy of *P. aeruginosa* chronic lung infections, despite showing irrelevant antibiotic activity on *P. aeruginosa* [88]. The antivirulence activity of azithromycin is still poorly understood, also because it is accompanied by other pharmacological properties, primarily the anti-inflammatory activity. However, evidence has been provided that azithromycin may selectively affect the translation of distinct subsets of *P. aeruginosa* genes depending on their codon usage, including the *rhlR* gene [89]. Moreover, among virulence phenotypes most strictly dependent on PqsE functionality, pyocyanin production, swarming motility and biofilm formation appear strongly affected by both azithromycin [88,89] and erythromycin estolate.

Overall, both nitrofuran and macrolides antibiotic activity is related to ribosome inhibition, raising the possibility that at low (sub-MIC) concentrations, these drugs could selectively affect the expression of PqsE-dependent genes at the translational level. Intriguingly, the presence of a ssDNA- or RNA-binding domain has been detected in PqsE [38,82], even if involvement of this domain in PqsE function has not yet been demonstrated.

The transfer of a new antivirulence drug to the clinical use will require the assessment of potential interactions with other drugs. Interestingly, neither nitrofurazone, nor erythromycin estolate had antagonistic effects toward antibiotics commonly used to treat *P. aeruginosa* infections. Moreover, *pqsE* deletion halved *P. aeruginosa* MIC values for tobramycin in M9-Glu, while this effect was absent in MHB. This indicates that PqsE could positively contribute to *P. aeruginosa* resistance to tobramycin depending on the growth medium, in agreement with studies showing that PqsE-dependent virulence genes are differentially regulated depending on the growth condition [40,41]. Among the antibiotics tested, *pqsE* deletion affected only the susceptibility of *P. aeruginosa* to tobramycin, indicating that PqsE could be required for the expression of factors contributing to resistance to aminoglicosides. It is also interesting to highlight that, among the antibiotics tested, only tobramycin targets the ribosome, supporting the existence of a link between PqsE activity and ribosome activity. Surprisingly, only nitrofurazone disclosed a tobramycin potentiating activity. It is not easy to explain why erythromycin estolate did not show this activity, also considering that this drug showed an overall higher activity than nitrofurazone on other PqsE-dependent phenotypes. One plausible explanation is that, besides PqsE, erythromycin estolate could hit other targets, thus compensating the effect on tobramycin susceptibility.

Antibiotic tolerance is the capacity of bacterial sub-populations to tolerate exposure to lethal concentrations of bactericidal antibiotics and relies upon mechanisms different from antibiotic resistance [90]. Previous studies showed that PqsR inhibitors could affect *P. aeruginosa* antibiotic tolerance [46]. However, this effect seems to be PqsE-independent, since in our hands antibiotic tolerance was not affected by *pqsE* deletion. Nonetheless, erythromycin estolate showed a PqsE-independent positive effect on tolerance to tobramycin. This result further supports the hypothesis that erythromycin estolate could hit molecular targets other than PqsE.

To the best of our knowledge this is the first report demonstrating the contribution of PqsE-dependent regulation to antibiotic resistance and providing a proof of concept that a PqsE inhibitor, in addition to its antivirulence activity, can potentiate antibiotic effect. In line with previous data [46], our study highlights that distinct anti-QS drugs targeting the same molecular pathway could differentially alter antibiotic resistance and tolerance, an issue worth to be taken into account for the development of antivirulence drugs.

As anticipated, the use of QS inhibitors for the treatment of CF patients chronically infected with *P. aeruginosa* is under debate, mainly due to the high genotypic and phenotypic variability generated by within-host evolution of the infecting strain. Indeed, during years of chronic infection in the lung, clonal variants of the initial population are positively selected by the peculiar CF lung environment and continuative drug administration. *P. aeruginosa* phenotypic variants with high levels of biofilm formation, high resistance to antibiotics and decreased production of secreted virulence factors, often associated to mutations in the *las* QS systems, are frequently isolated from the CF lung [24–27]. This implies that the range of activity of a novel antivirulence drug developed against *P. aeruginosa* should be determined in a representative collection of CF isolates to predict its potential in therapy of CF infection. The experiments carried out in the final part of this study were not aimed at fully addressing this issue, but rather at preliminarily comparing nitrofurazone and erythromycin estolate activity in a small number of CF strains proficient in AQ and pyocyanin production, hence potentially susceptible to PqsE inhibitors. Our pilot tests indicate that both nitrofurazone and erythromycin estolate significantly decrease pyocyanin production in the majority of the tested CF isolates, and that erythromycin estolate displays a higher range of activity compared to nitrofurazone.

In the last years, our group proved the feasibility of the drug-repurposing approach based on wet-lab or virtual screenings for the identification of FDA-approved drugs targeting *P. aeruginosa* virulence [49,52,91,92]. This additional study provides the first example of target-oriented screening aimed at identifying FDA-approved inhibitors of PqsE-dependent virulence factors. The recovery of two hits with strong anti-PqsE activity in a library of 1,600 compounds validated this system, which is suitable for future high-throughput screening of larger compound libraries. The PqsE-inhibitors here identified are endowed with intrinsic antibiotic properties though they have no effect on *P. aeruginosa* growth at concentrations causing PqsE inhibition. Notably, also clofoctol, an antibiotic active against Gram-positive bacteria, has strong anti-PqsR and antivirulence activity at concentrations unable to affect *P. aeruginosa* growth [49]. These observations support the hypothesis that some antibiotics secreted at low concentration in a polymicrobial community could play a role as signaling molecules, rather than as inhibitors of competitors’ growth [93,94].

As antibiotics, nitrofurazone and erythromycin estolate can be predicted to have a significant impact on the host microbiota. Moreover, the intrinsic toxicity of both erythromycin estolate and nitrofurazone for eukaryotic cells could be a limitation for the repurposing of these drugs to treat *P. aeruginosa* infections. These limitations of the drug repurposing strategy for antivirulence therapy should not be overlooked, as previously outlined by our group [9]. On the other side, it should be taken into account that also a high proportion of non-antibiotic drugs can alter the gut microbiome composition [95], and that the erythromycin analogue azithromycin is already used as antivirulence drug, even if on an empirical basis [88]. In fact, the emergence of MDR and extensively drug-resistant bacteria is leading to the rediscovery and/or optimization of fallen into disuse drugs, in accordance to the selective optimization of side activities (SOSA) approach [96]. As an example, nitrofuran drugs with low toxicity against eukaryotic cells and high activity against *Mycobacterium tuberculosis* are under development [97].

Finally, beyond their possible applications to the therapy, a main result of this study is the identification of two compounds specifically targeting the expression of PqsE-dependent genes, which could provide useful tools for future studies aimed at unraveling the mechanism of action of PqsE.

## Supplementary Material

Supplemental Material

## References

[1] Martínez JL, Baquero F. Emergence and spread of antibiotic resistance: setting a parameter space. Ups J Med Sci. 2014;119:68–77. 24678768 10.3109/03009734.2014.901444 PMC4034563

[2] Payne DJ, Gwynn MN, Holmes DJ, et al. Drugs for bad bugs: confronting the challenges of antibacterial discovery. Nat Rev Drug Discov. 2007;6:29–40. 17159923 10.1038/nrd2201

[3] So AD, Gupta N, Brahmachari SK, et al. Towards new business models for R&D for novel antibiotics. Drug Resist Updat. 2011;14:88–94. 21439891 10.1016/j.drup.2011.01.006

[4] Ribeiro da Cunha B, Fonseca LP, Calado CRC. Antibiotic discovery: where have we come from, where do we go? Antibiotics (Basel). 2019;8:2. 10.3390/antibiotics8020045 PMC6627412 31022923

[5] Rangel-Vega A, Bernstein LR, Mandujano-Tinoco EA, et al. Drug repurposing as an alternative for the treatment of recalcitrant bacterial infections. Front Microbiol. 2015;6:282. 25914685 10.3389/fmicb.2015.00282 PMC4391038

[6] Monserrat-Martinez A, Gambin Y, Sierecki E. Thinking outside the bug: molecular targets and strategies to overcome antibiotic resistance. Int J Mol Sci. 2019;20:6. 10.3390/ijms20061255 PMC6470534 30871132

[7] Rasko DA, Sperandio V. Antivirulence strategies to combat bacteria-mediated disease. Drug Discovery. 2010;9:117–128. 20081869 10.1038/nrd3013

[8] Allen RC, Popat R, Diggle SP, et al. Targeting virulence: can we make evolution-proof drugs? Nat Rev Microbiol. 2014;12:300–308. 24625893 10.1038/nrmicro3232

[9] Rampioni G, Visca P, Leoni L, et al. Drug repurposing for antivirulence therapy against opportunistic bacterial pathogens. Emerg Top Life Sci. 2017;1:13–22. 33525812 10.1042/ETLS20160018

[10] Moradali MF, Ghods S, Rehm BHA. Pseudomonas aeruginosa lifestyle: a paradigm for adaptation, survival, and persistence. Front Cell Infect Microbiol. 2017;7:39. 28261568 10.3389/fcimb.2017.00039 PMC5310132

[11] Breidenstein EB, de la Fuente-núñez C, Hancock RE. Pseudomonas aeruginosa: all roads lead to resistance. Trends Microbiol. 2011;19(8):419–426. 21664819 10.1016/j.tim.2011.04.005

[12] Lyczak JB, Cannon CL, Pier GB. Establishment of Pseudomonas aeruginosa infection: lessons from a versatile opportunist. Microbes Infect. 2000;2:1051–1060. 10967285 10.1016/s1286-4579(00)01259-4

[13] Pendleton JN, Gorman SP, Gilmore BF. Clinical relevance of the ESKAPE pathogens. Expert Rev Anti Infect Ther. 2013;11:297–308. 23458769 10.1586/eri.13.12

[14] Poole K. Pseudomonas aeruginosa: resistance to the max. Front Microbiol. 2011;2:65. 21747788 10.3389/fmicb.2011.00065 PMC3128976

[15] Alcade-Rico M, Hernando-Amado S, Blanco P, et al. Multidrug efflux pumps at the crossroad between antibiotic resistance and bacterial virulence. Front Microbiol. 2016;7:1483. 27708632 10.3389/fmicb.2016.01483 PMC5030252

[16] Jimenez PN, Koch G, Thompson JA, et al. The multiple signaling systems regulating virulence in Pseudomonas aeruginosa. Microbiol Mol Biol Rev. 2012;76:46–65. 22390972 10.1128/MMBR.05007-11 PMC3294424

[17] Balasubramanian D, Schneper L, Kumari H, et al. A dynamic and intricate regulatory network determines Pseudomonas aeruginosa virulence. Nucleic Acids Res. 2013;41:1–20. 23143271 10.1093/nar/gks1039 PMC3592444

[18] Kirisits MJ, Parsek MR. Does Pseudomonas aeruginosa use intercellular signaling to build biofilm communities? Cell Microbiol. 2006;8:1841–1849. 17026480 10.1111/j.1462-5822.2006.00817.x

[19] Rutherford ST, Bassler BL. Bacterial quorum sensing: its role in virulence and possibilities for its control. Cold Spring Harb Perspect Med. 2012;2:a012427-a012427. 23125205 10.1101/cshperspect.a012427 PMC3543102

[20] LaSarre B, Federle MJ. Exploiting quorum sensing to confuse bacterial pathogens. Microbiol Mol Biol Rev. 2013;77:73–111. 23471618 10.1128/MMBR.00046-12 PMC3591984

[21] Rampioni G, Leoni L, Williams P. The art of antibacterial warfare: deception through interference with quorum sensing-mediated communication. Bioorg Chem. 2014;55:60–68. 24823895 10.1016/j.bioorg.2014.04.005

[22] Williams P, Cámara M. Quorum sensing and environmental adaptation in Pseudomonas aeruginosa: a tale of regulatory networks and multifunctional signal molecules. Curr Opin Microbiol. 2009;12:182–191. 19249239 10.1016/j.mib.2009.01.005

[23] Smith RS, Iglewski BH. P. aeruginosa quorum-sensing systems and virulence. Curr Opin Microbiol. 2003;6:56–60. 12615220 10.1016/s1369-5274(03)00008-0

[24] Hoffman LR, Kulasekara HD, Emerson J, et al. Pseudomonas aeruginosa lasR mutants are associated with cystic fibrosis lung disease progression. J Cyst Fibros. 2009;8:66–70. 18974024 10.1016/j.jcf.2008.09.006 PMC2631641

[25] Marvig RL, Sommer LM, Molin S, et al. Convergent evolution and adaptation of Pseudomonas aeruginosa within patients with cystic fibrosis. Nat Genet. 2015;47:57–64. 25401299 10.1038/ng.3148

[26] Feltner JB, Wolter DJ, Pope CE, et al. LasR variant cystic fibrosis isolates reveal an adaptable quorum-sensing hierarchy in Pseudomonas aeruginosa. MBio. 2016;7:5. 10.1128/mBio.01513-16 PMC5050340 27703072

[27] Winstanley C, O’Brien S, Brockhurst MA. Pseudomonas aeruginosa evolutionary adaptation and diversification in cystic fibrosis chronic lung infections. Trends Microbiol. 2016;24:327–337. 26946977 10.1016/j.tim.2016.01.008 PMC4854172

[28] Guina T, Purvine SO, Yi EC, et al. Quantitative proteomic analysis indicates increased synthesis of a quinolone by Pseudomonas aeruginosa isolates from cystic fibrosis airways. Proc Natl Acad Sci USA. 2003;100:2771–2776. 12601166 10.1073/pnas.0435846100 PMC151416

[29] Jiricny N, Molin S, Foster K, et al. Loss of social behaviours in populations of Pseudomonas aeruginosa infecting lungs of patients with cystic fibrosis. PLoS One. 2014;9:e83124. 24454693 10.1371/journal.pone.0083124 PMC3891558

[30] Barr HL, Halliday N, Cámara M, et al. Pseudomonas aeruginosa quorum sensing molecules correlate with clinical status in cystic fibrosis. Eur Respir J. 2015;46:1046–1054. 26022946 10.1183/09031936.00225214 PMC4589431

[31] Heeb S, Fletcher MP, Chhabra SR, et al. Quinolones: from antibiotics to autoinducers. FEMS Microbiol Rev. 2011;35:247–274. 20738404 10.1111/j.1574-6976.2010.00247.x PMC3053476

[32] Dulcey CE, Dekimpe V, Fauvelle DA, et al. The end of an old hypothesis: the Pseudomonas signaling molecules 4-hydroxy-2-alkylquinolines derive from fatty acids, not 3-ketofatty acids. Chem Biol. 2013;20:1481–1491. 24239007 10.1016/j.chembiol.2013.09.021 PMC3877684

[33] Drees SL, Fetzner S. PqsE of Pseudomonas aeruginosa acts as pathway-specific thioesterase in the biosynthesis of alkylquinolone signaling molecules. Chem Biol. 2015;22:611–618. 25960261 10.1016/j.chembiol.2015.04.012

[34] Rampioni G, Falcone M, Heeb S, et al. Unravelling the genome-wide contributions of specific 2-alkyl-4-quinolones and PqsE to quorum sensing in Pseudomonas aeruginosa. PLoS Pathog. 2016;12:e1006029. 27851827 10.1371/journal.ppat.1006029 PMC5112799

[35] Hazan R, He J, Xiao G, et al. Homeostatic interplay between bacterial cell-cell signaling and iron in virulence. PLoS Pathog. 2010;6:e1000810. 20300606 10.1371/journal.ppat.1000810 PMC2837411

[36] Rampioni G, Pustelny C, Fletcher MP, et al. Transcriptomic analysis reveals a global alkyl-quinolone-independent regulatory role for PqsE in facilitating the environmental adaptation of Pseudomonas aeruginosa to plant and animal hosts. Environ Microbiol. 2010;12:1659–1673. 20406282 10.1111/j.1462-2920.2010.02214.x PMC2901523

[37] Farrow JM 3rd, Sund ZM, Ellison ML, et al. PqsE functions independently of PqsR-Pseudomonas quinolone signal and enhances the rhl quorum-sensing system. J Bacteriol. 2008;190:7043–7051. 18776012 10.1128/JB.00753-08 PMC2580708

[38] Folch B, Déziel E, Doucet N. Systematic mutational analysis of the putative hydrolase PqsE: toward a deeper molecular understanding of virulence acquisition in Pseudomonas aeruginosa. PLoS ONE. 2013;8:e73727. 24040042 10.1371/journal.pone.0073727 PMC3769375

[39] Zender M, Witzgall F, Drees SL, et al. Dissecting the multiple roles of PqsE in Pseudomonas aeruginosa virulence by discovery of small tool compounds. ACS Chem Biol. 2016;11:1755–1763. 27082157 10.1021/acschembio.6b00156

[40] Mukherjee S, Moustafa DA, Stergioula V, et al. The PqsE and RhlR proteins are an autoinducer synthase-receptor pair that control virulence and biofilm development in Pseudomonas aeruginosa. Proc Natl Acad Sci USA. 2018;115:E9411–E9418. 30224496 10.1073/pnas.1814023115 PMC6176596

[41] McCready AR, Paczkowski JE, Cong JP, et al. An autoinducer-independent RhlR quorum-sensing receptor enables analysis of RhlR regulation. PLoS Pathog. 2019;15:e1007820. 31194839 10.1371/journal.ppat.1007820 PMC6564026

[42] Klein T, Henn C, de Jong JC, et al. Identification of small-molecule antagonists of the Pseudomonas aeruginosa transcriptional regulator PqsR: biophysically guided hit discovery and optimization. ACS Chem Biol. 2012;7:1496–1501. 22765028 10.1021/cb300208g

[43] Ilangovan A, Fletcher M, Rampioni G, et al. Structural basis for native agonist and synthetic inhibitor recognition by the Pseudomonas aeruginosa quorum sensing regulator PqsR (MvfR). PLoS Pathog. 2013;9:e1003508. 23935486 10.1371/journal.ppat.1003508 PMC3723537

[44] Zender M, Klein T, Henn C, et al. Discovery and biophysical characterization of 2-amino-oxadiazoles as novel antagonists of PqsR, an important regulator of Pseudomonas aeruginosa virulence. J Med Chem. 2013;56:6761–6774. 23919758 10.1021/jm400830r

[45] Lu C, Kirsch B, Maurer CK, et al. Optimization of anti-virulence PqsR antagonists regarding aqueous solubility and biological properties resulting in new insights in structure-activity relationships. Eur J Med Chem. 2014;79:173–183. 24735643 10.1016/j.ejmech.2014.04.016

[46] Starkey M, Lepine F, Maura D, et al. Identification of anti-virulence compounds that disrupt quorum-sensing regulated acute and persistent pathogenicity. PLoS Pathog. 2014;10:e1004321. 25144274 10.1371/journal.ppat.1004321 PMC4140854

[47] Maura D, Drees SL, Bandyopadhaya A, et al. Polypharmacology approaches against the Pseudomonas aeruginosa MvfR regulon and their application in blocking virulence and antibiotic tolerance. ACS Chem Biol. 2017;12:1435–1443. 28379691 10.1021/acschembio.6b01139 PMC12908516

[48] Maura D, Rahme LG. Pharmacological inhibition of the Pseudomonas aeruginosa MvfR quorum-sensing system interferes with biofilm formation and potentiates antibiotic-mediated biofilm disruption. Antimicrob Agents Chemother. 2017;61. DOI:10.1128/AAC.01362-17 PMC5700327 28923875

[49] D’Angelo F, Baldelli V, Halliday N, et al. Identification of FDA-approved drugs as antivirulence agents targeting the pqs quorum-sensing system of Pseudomonas aeruginosa. Antimicrob Agents Chemother. 2018;62. DOI:10.1128/AAC.01296-18. PMC6201120 30201815

[50] Soukarieh F, Williams P, Stocks MJ, et al. Pseudomonas aeruginosa quorum sensing systems as drug discovery targets: current position and future perspectives. J Med Chem. 2018a;61:10385–10402. 29999316 10.1021/acs.jmedchem.8b00540

[51] Soukarieh F, Vico Oton E, Dubern JF, et al. In silico and in vitro-guided identification of inhibitors of alkylquinolone-dependent quorum sensing in Pseudomonas aeruginosa. Molecules. 2018b;23:257. 10.3390/molecules23020257 PMC6017655 29382099

[52] Mellini M, Di Muzio E, D’Angelo F, et al. In silico selection and experimental validation of FDA-approved drugs as anti-quorum sensing agents. Front Microbiol. 2019;10:2355. 31649658 10.3389/fmicb.2019.02355 PMC6796623

[53] Grant SG, Jessee J, Bloom FR, et al. Differential plasmid rescue from transgenic mouse DNAs into Escherichia coli methylation-restriction mutants. Proc Natl Acad Sci USA. 1990;87:4645–4649. 2162051 10.1073/pnas.87.12.4645 PMC54173

[54] Sambrook J, Fritsch EF, Maniatis T. Molecular cloning: a laboratory manual. 2nd ed. New York: Cold Spring Harbor Laboratory Press, Cold Spring Harbor; 1989.

[55] Massai F, Imperi F, Quattrucci S, et al. A multitask biosensor for micro-volumetric detection of N-3-oxo-dodecanoyl-homoserine lactone quorum sensing signal. Biosens Bioelectron. 2011;26:3444–3449. 21324665 10.1016/j.bios.2011.01.022

[56] Essar DW, Eberly L, Hadero A, et al. Identification and characterization of genes for a second anthranilate synthase in Pseudomonas aeruginosa: interchangeability of the two anthranilate synthases and evolutionary implications. J Bacteriol. 1990;172:884–900. 2153661 10.1128/jb.172.2.884-900.1990 PMC208517

[57] Wilhelm S, Gdynia A, Tielen P, et al. The autotransporter esterase EstA of Pseudomonas aeruginosa is required for rhamnolipid production, cell motility, and biofilm formation. J Bacteriol. 2007;189:6695–6703. 17631636 10.1128/JB.00023-07 PMC2045186

[58] Rampioni G, Schuster M, Greenberg EP, et al. Contribution of the RsaL global regulator to Pseudomonas aeruginosa virulence and biofilm formation. FEMS Microbiol Lett. 2009;301:210–217. 19878323 10.1111/j.1574-6968.2009.01817.x

[59] Davies DG, Parsek MR, Pearson JP, et al. The involvement of cell-to-cell signals in the development of a bacterial biofilm. Science. 1998;280:295–298. 9535661 10.1126/science.280.5361.295

[60] Jurcisek JA, Dickson AC, Bruggeman ME, et al. In vitro biofilm formation in an 8-well chamber slide. J Vis Exp. 2011;47:e2481. 10.3791/2481 PMC3182645 21304464

[61] CLSI. M07-A9. Clinical and Laboratory Standards Institute. Methods for dilution antimicrobial susceptibility tests for bacteria that grow aerobically. 9th ed. Wayne PA: CLSI (Approved standard M07-A9); 2012.

[62] Cramer DL, Dodd MC. The mode of action of nitrofuran compounds; action versus Staphylococcus aureus. J Bacteriol. 1946;51:293–303. 10.1128/JB.51.3.293-303.1946 21018705

[63] Lacey RW. A new look at erythromycin. Postgrad Med J. 1977;53:195–200. 323837 10.1136/pgmj.53.618.195 PMC2496485

[64] Schweizer HP. Escherichia-Pseudomonas shuttle vectors derived from pUC18/19. Gene. 1991;97:109–121. 1899844 10.1016/0378-1119(91)90016-5

[65] Higgins S, Heeb S, Rampioni G, et al. Differential regulation of the phenazine biosynthetic operons by quorum sensing in Pseudomonas aeruginosa PAO1-N. Front Cell Infect Microbiol. 2018;8:252. 30083519 10.3389/fcimb.2018.00252 PMC6064868

[66] Lau GW, Hassett DJ, Ran H, et al. The role of pyocyanin in Pseudomonas aeruginosa infection. Trends Mol Med. 2004;10:599–606. 15567330 10.1016/j.molmed.2004.10.002

[67] Papenfort K, Bassler BL. Quorum sensing signal-response systems in Gram-negative bacteria. Nat Rev Microbiol. 2016;14:576–588. 27510864 10.1038/nrmicro.2016.89 PMC5056591

[68] Martínez E, Cosnahan RK, Wu M, et al. Oxylipins mediate cell-to-cell communication in Pseudomonas aeruginosa. Commun Biol. 2019;2:66. 30793044 10.1038/s42003-019-0310-0 PMC6377657

[69] Cao H, Krishnan G, Goumnerov B, et al. A quorum sensing-associated virulence gene of Pseudomonas aeruginosa encodes a LysR-like transcription regulator with a unique self-regulatory mechanism. Proc Natl Acad Sci USA. 2001;98:14613–14628. 11724939 10.1073/pnas.251465298 PMC64730

[70] Diggle SP, Winzer K, Chhabra SR, et al. The Pseudomonas aeruginosa quinolone signal molecule overcomes the cell density-dependency of the quorum sensing hierarchy, regulates rhl-dependent genes at the onset of stationary phase and can be produced in the absence of LasR. Mol Microbiol. 2003;50:29–43. 14507361 10.1046/j.1365-2958.2003.03672.x

[71] De´ziel E, Gopalan S, Tampakaki AP, et al. The contribution of MvfR to Pseudomonas aeruginosa pathogenesis and quorum sensing circuitry regulation: multiple quorum sensing-regulated genes are modulated without affecting lasRI, rhlRI or the production of N-acyl-L-homoserine lactones. Mol Microbiol. 2005;55:998–1014. 15686549 10.1111/j.1365-2958.2004.04448.x

[72] Xiao G, Déziel E, He J, et al. MvfR, a key Pseudomonas aeruginosa pathogenicity LTTR-class regulatory protein, has dual ligands. Mol Microbiol. 2006;62:1689–1699. 17083468 10.1111/j.1365-2958.2006.05462.x

[73] Lesic B, Lépine F, Déziel E, et al. Inhibitors of pathogen intercellular signals as selective anti-infective compounds. PLoS Pathog. 2007;3:1229–1239. 17941706 10.1371/journal.ppat.0030126 PMC2323289

[74] Dubern JF, Cigana C, De Simone M, et al. Integrated whole-genome screening for Pseudomonas aeruginosa virulence genes using multiple disease models reveals that pathogenicity is host specific. Environ Microbiol. 2015;17:4379–4393. 25845292 10.1111/1462-2920.12863 PMC4676916

[75] Lau GW, Goumnerov BC, Walendziewicz CL, et al. The Drosophila melanogaster toll pathway participates in resistance to infection by the Gram-negative human pathogen Pseudomonas aeruginosa. Infect Immun. 2003;71:4059–4066. 12819096 10.1128/IAI.71.7.4059-4066.2003 PMC162001

[76] Maura D, Hazan R, Kitao T, et al. Evidence for direct control of virulence and defense gene circuits by the Pseudomonas aeruginosa quorum sensing regulator, MvfR. Sci Rep. 2016;6:34083. 27678057 10.1038/srep34083 PMC5039717

[77] Huang H, Shao X, Xie Y, et al. An integrated genomic regulatory network of virulence-related transcriptional factors in Pseudomonas aeruginosa. Nat Commun. 2019;10:2931. 31270321 10.1038/s41467-019-10778-w PMC6610081

[78] Mashburn LM, Whiteley M. Membrane vesicles traffic signals and facilitate group activities in a prokaryote. Nature. 2005;437:422–425. 16163359 10.1038/nature03925

[79] Bredenbruch F, Geffers R, Nimtz M, et al. The Pseudomonas aeruginosa quinolone signal (PQS) has an iron-chelating activity. Environ Microbiol. 2006;8:1318–1329. 16872396 10.1111/j.1462-2920.2006.01025.x

[80] Diggle SP, Matthijs S, Wright VJ, et al. The Pseudomonas aeruginosa 4-quinolone signal molecules HHQ and PQS play multifunctional roles in quorum sensing and iron entrapment. Chem Biol. 2007;14:87–96. 17254955 10.1016/j.chembiol.2006.11.014

[81] Lin J, Zhang W, Cheng J, et al. A Pseudomonas T6SS effector recruits PQS-containing outer membrane vesicles for iron acquisition. Nat Commun. 2017;8:14888. 28348410 10.1038/ncomms14888 PMC5379069

[82] Yu S, Jensen V, Seeliger J, et al. Structure elucidation and preliminary assessment of hydrolase activity of PqsE, the Pseudomonas quinolone signal (PQS) response protein. Biochemistry. 2009;48:10298–10307. 19788310 10.1021/bi900123j

[83] Sharma S, Anand N. Approaches to design and synthesis of antiparasitic drugs. Pharmacochemistry Lib. 1997;25:421–438.

[84] Vass M, Hruska K, Franek M. Nitrofuran antibiotics: a review on the application, prohibition and residual analysis. Vet Med. 2008;53:469–500.

[85] Olive PL, McCalla DR. Cytotoxicity and DNA damage to mammalian cells by nitrofurans. Chem Biol Interact. 1977;16:223–233. 849625 10.1016/0009-2797(77)90131-4

[86] McOsker CC, Fitzpatrick PM. Nitrofurantoin: mechanism of action and implications for resistance development in common uropathogens. J Antimicrob Chemother. 1994;33:23–30. 7928834 10.1093/jac/33.suppl_a.23

[87] Yang L, Rybtke MT, Jakobsen TH, et al. Computer-aided identification of recognized drugs as Pseudomonas aeruginosa quorum-sensing inhibitors. Antimicrob Agents Chemother. 2009;53:2432–2443. 19364871 10.1128/AAC.01283-08 PMC2687250

[88] Imperi F, Leoni L, Visca P. Antivirulence activity of azithromycin in Pseudomonas aeruginosa. Front Microbiol. 2014;5. DOI:10.3389/fmicb.2014.00178 24795709 PMC4001013

[89] Gödeke J, Pustelny C, Häussler S. Recycling of peptidyl-tRNAs by peptidyl-tRNA hydrolase counteracts azithromycin-mediated effects on Pseudomonas aeruginosa. Antimicrob Agents Chemother. 2013;57:1617–1624. 23318806 10.1128/AAC.02582-12 PMC3623356

[90] Balaban NQ, Helaine S, Lewis K, et al. Definitions and guidelines for research on antibiotic persistence. Nat Rev Microbiol. 2019;17:441–448. 30980069 10.1038/s41579-019-0196-3 PMC7136161

[91] Imperi F, Massai F, Facchini M, et al. Repurposing the antimycotic drug flucytosine for suppression of Pseudomonas aeruginosa pathogenicity. Proc Natl Acad Sci USA. 2013;110:7458–7463. 23569238 10.1073/pnas.1222706110 PMC3645532

[92] Imperi F, Massai F, Ramachandran Pillai C, et al. New life for an old drug: the anthelmintic drug niclosamide inhibits Pseudomonas aeruginosa quorum sensing. Antimicrob Agents Chemother. 2013;57:996–1005. 23254430 10.1128/AAC.01952-12 PMC3553739

[93] Davies J, Spiegelman GB, Yim G. The world of subinhibitory antibiotic concentrations. Curr Opin Microbiol. 2006;9:445–453. 16942902 10.1016/j.mib.2006.08.006

[94] Yim G, Wang HH, Davies J. Antibiotics as signalling molecules. Philos Trans R Soc Lond B Biol Sci. 2007;362:1195–1200. 17360275 10.1098/rstb.2007.2044 PMC2435582

[95] Maier L, Pruteanu M, Kuhn M, et al. Extensive impact of non-antibiotic drugs on human gut bacteria. Nature. 2018;555:623–628. 29555994 10.1038/nature25979 PMC6108420

[96] Wermuth CG. Selective optimization of side activities: the SOSA approach. Drug Discov Today. 2006;11:160–164. 16533714 10.1016/S1359-6446(05)03686-X

[97] Elsaman T, Mohamed MS, Mohamed MA. Current development of 5-nitrofuran-2-yl derivatives as antitubercular agents. Bioorg Chem. 2019;88:102969. 31077910 10.1016/j.bioorg.2019.102969

