# Supplementary material for: Identification of FDA-approved antivirulence drugs targeting the Pseudomonas aeruginosa quorum sensing effector protein PqsE
Source: Virulence. 2020 May 28;11(1):652–68. doi: 10.1080/21505594.2020.1770508 (PMC7549961; doi:10.1080/21505594.2020.1770508)

Identification of FDA-approved antivirulence drugs targeting the *Pseudomonas aeruginosa* quorum sensing effector protein PqsE

Valerio Baldelli[a], Francesca D'Angelo[a#], Viola Pavoncello[a#], Ersilia Vita Fiscarelli[b], Paolo Visca[b], Giordano Rampioni[a*], Livia Leoni[a*]

[a] Department of Science, University Roma Tre, Rome, Italy; [b] Laboratory of Cystic Fibrosis Microbiology, Bambino Gesú Hospital, Rome, Italy.

[#] Current address: Institut Pasteur, Paris, France.

SUPPLEMENTAL MATERIAL

Table S1. Strains used in this study

Table S2. Plasmids used in this study

Table S3. MIC of selected antibiotics

Figure S1. Set up of the PqsE-Rep biosensor system

Figure S2. Primary and secondary screens of the PHARMAKON library

Figure S3. Growth curves of *P. aeruginosa* in the presence of PqsE inhibitors

Figure S4. Effect of the PqsE inhibitors on constitutive bioluminescence

Figure S5. Effect of PqsE inhibitors on *P. aeruginosa* tolerance to tobramycin

Table S1. Strains used in this study

Strain	Description	References
PAO1	wild type strain.	ATCC15692
PAO1 Δ*pqsE*	PAO1 derivative carrying an in-frame deletion of the *pqsE* gene.	[36]
PAO1 PqsE-Rep (*pqsE*$_{IND}$ P*pqsA*::*lux*)	PAO1 derivative in which *pqsE* expression is IPTG inducible and containing the P*pqsA*::*luxCDABE* transcriptional fusion integrated into the chromosome at the *attB* neutral site; Tc^{R}.	[36]
PAO1 P*pqsA::lux*	PAO1 derivative containing the P*pqsA*::*luxCDABE* transcriptional fusion integrated into the chromosome at the *attB* neutral site; Tc^{R}.	[98]
PAO1 Δ*pqsE* P*pqsA*::*lux*	PAO1 Δ*pqsE* derivative containing the P*pqsA*::*luxCDABE* transcriptional fusion integrated into the chromosome at the *attB* neutral site; Tc^{R}.	[36]
PAO1 mini-CTX-*lux*	PAO1 derivative containing the mini-CTX-*lux* empty vector integrated into the chromosome at the *attB* neutral site; Tc^{R}.	[98]
PAO1 Δ*pqsE* mini-CTX-*lux*	PAO1 Δ*pqsE* derivative containing the mini-CTX-*lux* empty vector integrated into the chromosome at the *attB* neutral site; Tc^{R}.	[36]

Table S2. Plasmids used in this study

Plasmid	Relevant characteristics	References
pUCP18	pUC18-derivative containing a stabilising fragment for maintenance in *Pseudomonas* spp.; Ap^R, *E. coli*/Cb^R, *P. aeruginosa*.	[64]
pUCP-*pqsE*	pUCP18 derivative for *pqsE* complementation; Ap^R, *E. coli*/Cb^R, *P. aeruginosa*.	[36]
pMRP9-1	pUC18 derivative allowing constitutive expression of the *Aequorea victoria* GFP protein; Cb^R.	[59]
mini-CTX-*lux*	Promoter-probe vector containing the *luxCDABE* operon as reporter system; Tc^R.	[99]
mini-CTX-P*pqsA*::*lux*	mini-CTX-*lux* derivative used for the insertion of the P*pqsA*::*luxCDABE* transcriptional fusion into PAO1 chromosome; Tc^R.	[80]

References not included in the main text

[98] Fletcher MP, Diggle SP, Crusz SA, et al. A dual biosensor for 2-alkyl-4-quinolone quorum-sensing signal molecules. Environ Microbiol. 2007;9:2683-2693.

[99] Becher A, Schweizer HP. Integration-proficient *Pseudomonas aeruginosa* vectors for isolation of single-copy chromosomal *lacZ* and *lux* gene fusions. Biotechniques. 2000;29:948-950.

Table S3. MIC of selected antibiotics

	**Ciprofloxacin**		**Colistin**		**Tobramycin**		**Piperacillin**	
**Strain**	**MHB**	**M9**	**MHB**	**M9**	**M**	**M9**	**MH**	**M9**
P. aeruginosa PAO1	0.125	0.03125	2	4	0.5	0.5	8	2
P. aeruginosa Δ*pqsE*	0.125	0.03125	2	4	0.5	0.25	8	2

Figure S1. Set up of the PqsE-Rep biosensor system

(A) Activity of the P*pqsA* promoter in the PqsE-Rep strain grown in LB supplemented with the indicated concentrations of IPTG, after 3 h (white bars), 5 h (light-grey bars) and 7 h (dark-grey bars) of incubation at 37°C. **(B)** Activity of the P*pqsA* promoter in the PqsE-Rep strain inoculated at starting optical density (OD_{600}) of 0.08 (white bars), 0.03 (light-grey bars) and 0.01 (dark-grey bars), after 5 h of incubation at 37°C in LB supplemented with the indicated concentrations of IPTG. **(C)** Activity of the P*pqsA* promoter in the PqsE-Rep strain inoculated at a starting OD_{600} of 0.08 after 5 h of incubation in LB (white bars) or in LB supplemented with 50 µM IPTG (grey bars) at 30°C or 37°C, in static or shaking (120 rpm) conditions. For **(A)**-**(C)**, biosensor activity is reported as relative light units (RLU) normalized to cell density (OD_{600}); the average of three independent experiments is reported with SD.

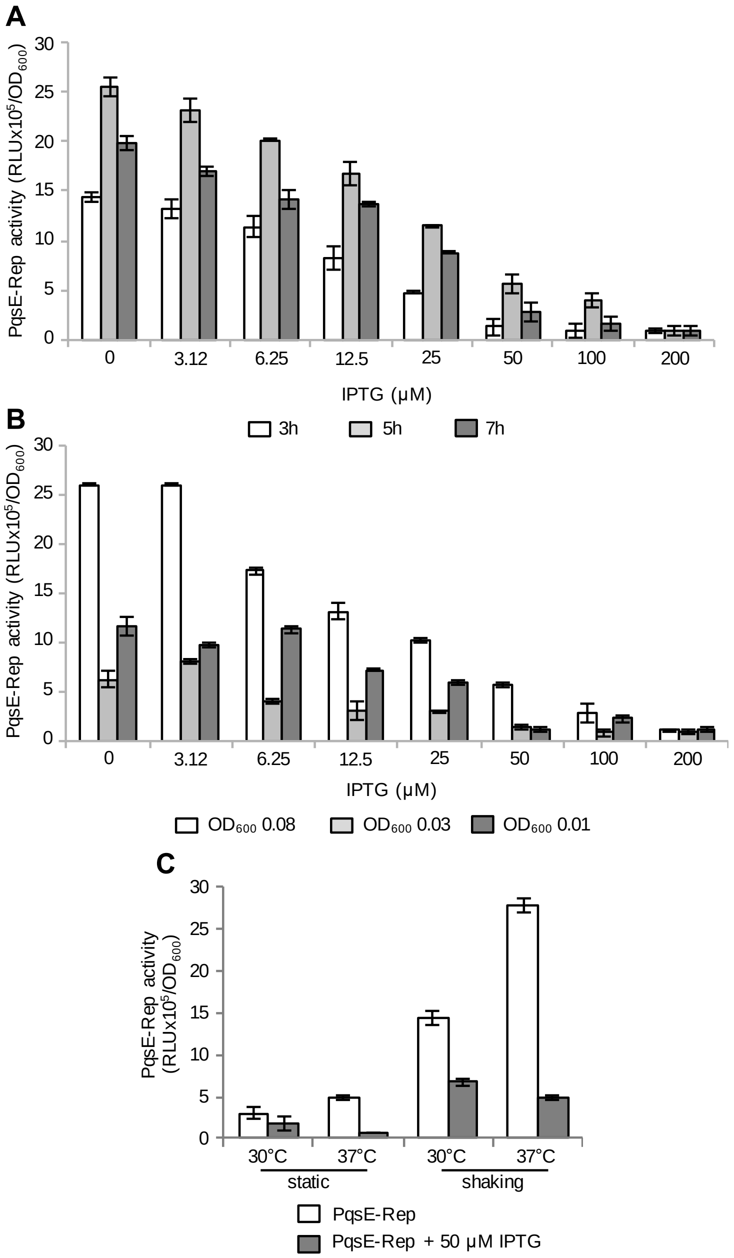

Figure S2. Primary and secondary screens of the PHARMAKON library

(A) Activity of the P*pqsA* promoter (bars) and cell density (diamonds) measured in the PqsE-Rep strain after 5 h incubation at 37°C in shaking conditions in LB supplemented with 50 µM IPTG and with the molecules of the PHARMAKON library, indicated with codes from inhibitor 1 (I-1) to inhibitor 24 (I-24), at 20 µM (white bars and diamonds) or 200 µM (grey bars and diamonds) concentration. PqsE-Rep activity and cell density measured in the presence of 0.2% (v/v) and 2% (v/v) DMSO were considered as 100%, respectively. **(B)** Pyocyanin production measured in supernatants of the PqsE-Rep biosensor strain supplemented with 50 µM IPTG and treated with the PHARMAKON library compounds nitrofurazone (I-2), erythromycin estolate (I-3) and diminazene aceturate (I-8) at 20 µM (white bars) and 200 µM (grey bars) concentration.

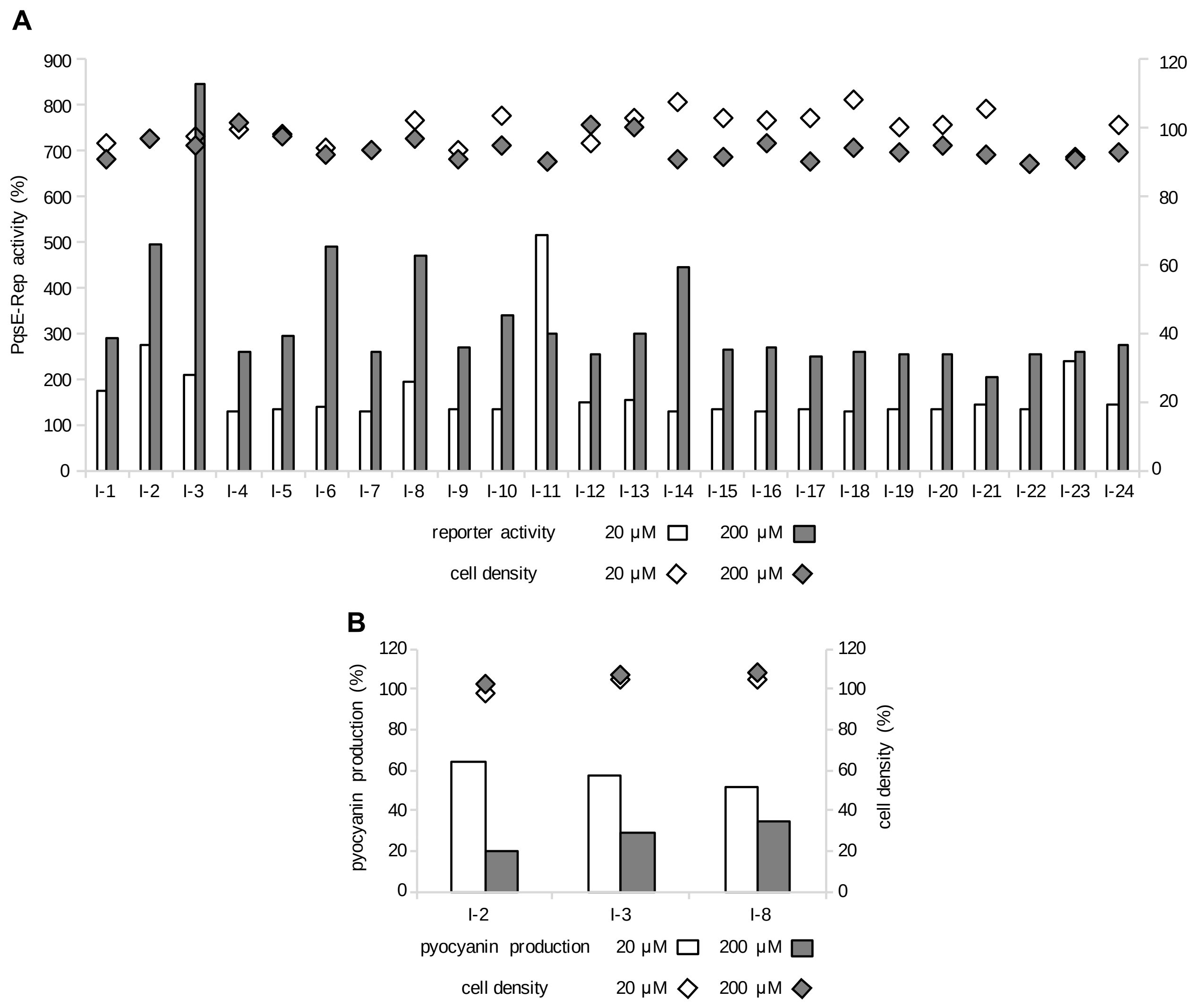

Figure S3. Growth curves of *P. aeruginosa* in the presence of PqsE inhibitors

Growth curves of *P. aeruginosa* PAO1 and its isogenic Δ*pqsE* mutant incubated at 37°C in shaking conditions in LB supplemented with: **(A)** 100 µM nitrofurazone (PAO1, blue; PAO1 Δ*pqsE,* black) or 0.125% (v/v) DMSO (PAO1, red; PAO1 Δ*pqsE,* green); **(B)** 50 µM erythromycin estolate (PAO1, blue; PAO1 Δ*pqsE,* black), or 0.025% (v/v) EtOH (PAO1, red; PAO1 Δ*pqsE,* green). The average of three independent experiments is reported with SD.

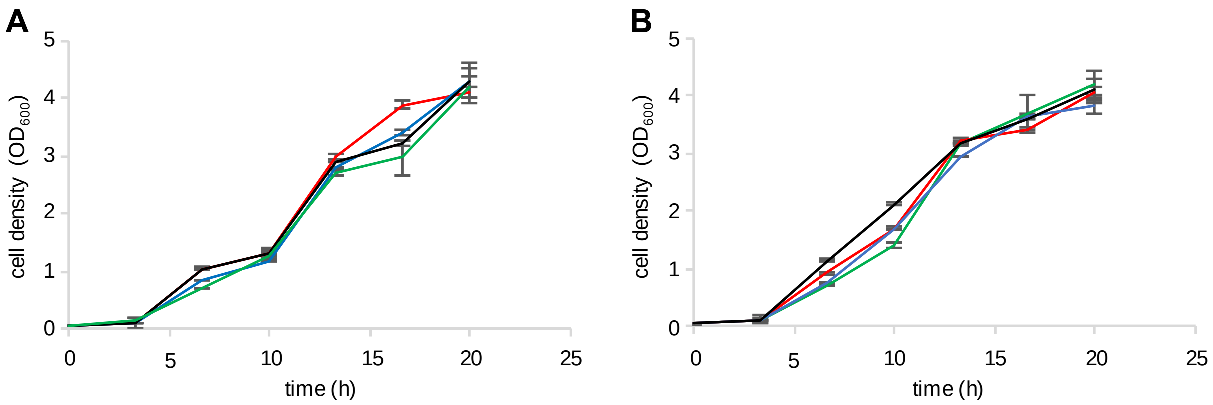

Figure S4. Effect of the PqsE inhibitors on constitutive bioluminescence

Percentage of light emitted by the indicated *P. aeruginosa* PAO1 strains carrying the mini-CTX-*lux* empty vector. The strains were grown at 37°C in shaking conditions in LB supplements with 100 µM nitrofurazone **(A)** or 50 µM erythromycin estolate **(B)**. Bioluminescence emitted by the same strains grown in the presence of 0.125% (v/v) DMSO or 0.025% (v/v) EtOH was considered as 100%. The average of three independent experiments is reported with SD.

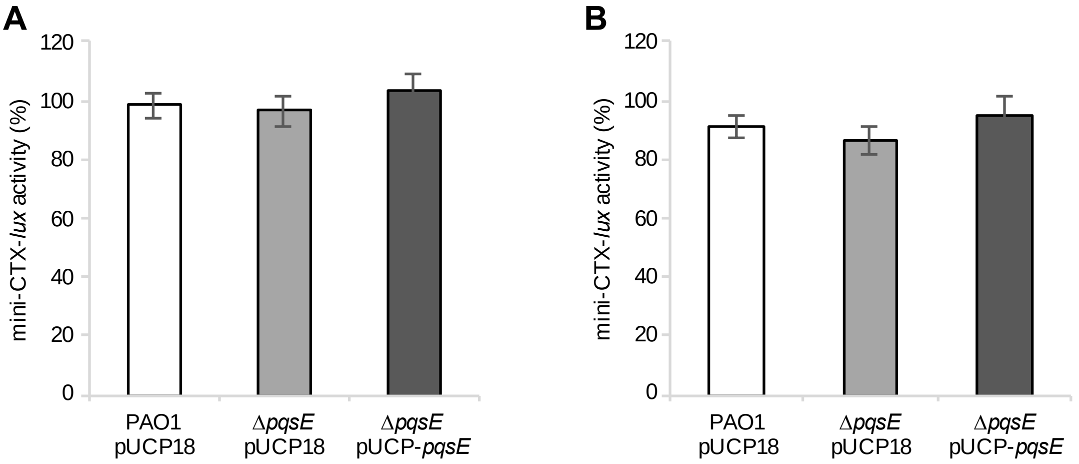

Figure S5. Effect of PqsE inhibitors on *P. aeruginosa* tolerance to tobramycin

Fraction of *P. aeruginosa* PAO1 cells tolerant to 4 µg/mL tobramycin (8x MIC) untreated (white bar) or after the treatment with 100 µM nitrofurazone (light-grey bar) or 50 µM erythromycin estolate (dark-grey bar). The untreated PAO1 Δ*pqsE* strain was used as control (black bar). The tolerant fraction expressed as N-fold change was determined as the ratio between the CFU/mL values measured after antibiotic addition (24 h post-antibiotic) divided by CFU/mL values measured before antibiotic addition. The average of three independent experiments is reported with SD. Similar results were obtained 16 h post-antibiotic treatment.

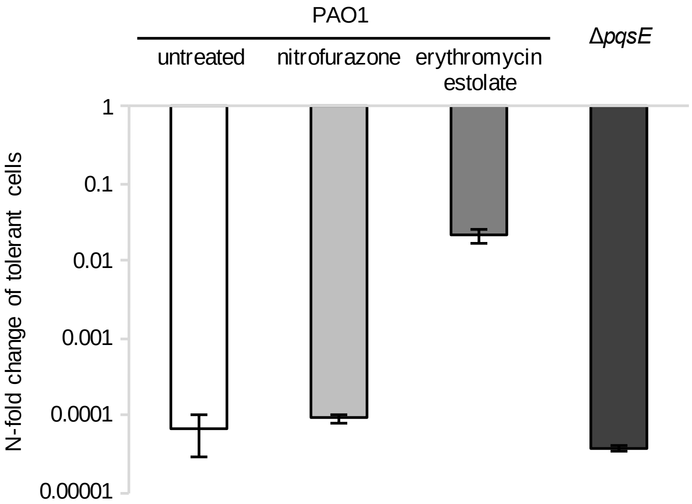

Supplement: Supplemental Material [file KVIR_A_1770508_SM9491.pdf]
